# Bright Zinc Probes with Thiomorpholine Monoxide Auxochromes for Imaging Insulin Secretion

**DOI:** 10.1021/cbmi.4c00116

**Published:** 2025-03-04

**Authors:** Rundong Yu, Junwei Zhang, Xiaohong Peng, Zhixing Chen

**Affiliations:** † State Key Laboratory of Membrane Biology, Institute of Molecular Medicine, National Biomedical Imaging Center, Beijing Key Laboratory of Cardiometabolic Molecular Medicine, College of Future Technology, 12465 Peking University, Beijing 100871, China; ‡ Peking-Tsinghua Center for Life Sciences, Academy for Advanced Interdisciplinary Studies, Peking University, Beijing 100871, China; ∇ School of Basic Medical Sciences, Shenzhen University Medical School, Shenzhen University, Shenzhen 518060, China; ∥ Department of Physiology and Pathophysiology, School of Basic Medical Sciences, Peking University, Beijing 100191, China

**Keywords:** Fluorescent Probe, Zinc, TICT, Islet Physiology, Insulin Secretion, Biocompatibility

## Abstract

Zinc biology significantly impacts human physiological and pathological processes, especially for β-cell endocrinology. In 2021, our group reported the **PKZnR** family with minimal phototoxicity and μM affinities for monitoring Zn^2+^/insulin corelease during vesicular secretory events on β-cells (Zhang et al. Angew. Chem. Int. Ed. 2021, 60 (49), 25846−25855 10.1002/anie.20210951034423531). Here, we synthesized a series of Zn^2+^ probes (**PKZnBR**) featuring thiomorpholine monoxide auxochromes with *K*_d_ values ranging from 160 nM to 94 μM. By inhibiting the twisted intramolecular charge transfer (TICT) state, the fluorescence quantum yields of **PKZnBRs** can be effectively increased to ∼7 times those of **PKZnRs**. A privileged candidate, **PKZnBR-3**, has a high turn-on ratio (∼134), appropriate affinity (340 nM), and excellent hydrophilicity, making it a powerful tool for long-term *ex vivo* recording of insulin secretion in mouse islets with minimal phototoxicity.

## Graphical abstract

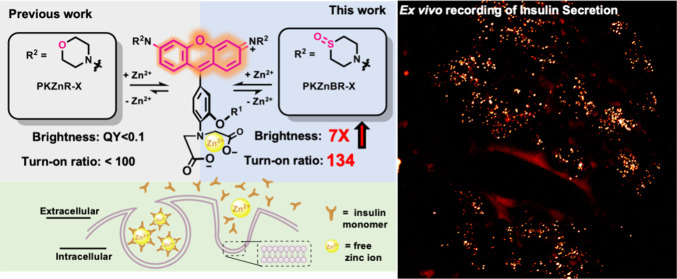

## Introduction

Zinc (Zn^2+^) impacts human health through its involvement in metabolic and immune processes. The concentration of Zn^2+^ exhibits high cellular heterogeneity and rapid dynamics, making fluorescent probes an irreplaceable tool for studying Zn^2+^ biology.^1^ Recently, the coevolution of fluorescent probes, microscopy, and image algorithms allowed us to obtain high-resolution spatiotemporal information at cellular or subcellular levels.^2,3^ Small-molecule ion probes have unique advantages in expanding the cutting edge in Zn^2+^ biology, as they can provide better photophysical properties in brightness and photostability than fluorescent protein and produce fluorescence signals with high spatiotemporal dynamics in living cells and tissues without genetic manipulation.^4–7^

The application of small-molecule-based probes in Zn^2+^ biology can be tracked to the 1960s when 8-hydroxyquinoline was used to quantitatively analyze Zn^2+^ in human serum or urine.^8^ Two decades later, another quinoline-based Zn^2+^ probe **TSQ** was synthesized with enhanced photophysical properties.^9^ Upon the half-century’s development, large quantities of small-molecule-based Zn^2+^ probes have been reported and can be divided into three types according to their activation mechanism. The first type is ICT (Intramolecular Charge Transfer)-based probes, such as the original version of **FuraZin** and **IndoZin** in the UV range and newly improved **NBD-TPE**, etc. in the visible region, featured on the ratiometric response of Zn^2+^.^10–16^ Another working mode known as FRET (Fluorescence Resonance Energy Transfer) bred a series of probes such as **CZ-1**, **CPBT**, etc.^17–20^ FRET-based Zn^2+^ probes were designed with paired fluorophores coupled with a short linker to ensure a higher FRET efficiency. The above two types of ratiomatric and intensiometric probes can be used for absolute quantification of Zn^2+^ concentration. Intensity-based probes usually sense Zn^2+^ through the PET (Photoinduced Electron Transfer) mechanism.^21,22^ For example, the fluorescein-based **QZ** probes and **ZP** probes were reported in the 2000s. Both of them exhibited high brightness and visible emission spectra.^23–25^ Meanwhile, derived from the DPA (2,2′-dipicolylamine) chelating group, the **ZnAF** probes also featured excellent selectivity and strong binding affinity toward Zn^2+^.^26–28^ In addition, another series of Zn^2+^ probes such as **RhodZin** and **FluoZin** were commercialized during the same period.^10,29^ Their structures are featured on a modified Ca^2+^ chelator BAPTA (1,2-bis­(2-aminophenoxy)­ethane-*N*,*N*,*N*′,*N*′-tetraacetic acid). These types of molecules can also be combined with a self-labeling protein tag to achieve precise subcellular localization.^30,31^ To date, chemists continue to evolve fluorophores and chelating groups to achieve higher brightness, faster kinetics, better selectivity, and tunable binding affinities (*K*_d_) for the growing Zn^2+^ biological research.

The remarkably high concentration of Zn^2+^ in insulin secretory granules (∼20 mM) suggests a special biological process. Insulin is vitally important for glucose homeostasis by lowering blood sugar levels. The crystal structure of insulin suggested that the coexistence of Zn^2+^ could stabilize the insulin hexamer structure.^32^ Basically, insulin is synthesized and stored in pancreatic β-cells and released as a Zn^2+^-insulin complex when blood glucose rises.^33,34^ During the diffusion of these complexes, the located transient concentration of Zn^2+^ could arrive at the level of micromole per liter.^35^ Thus, a plausible design for sensing insulin release is to detect the corelease of Zn^2+^ with fluorescent probes. In the 2010s, a series of fluorescein-based Zn^2+^ probes such as **ZIMIRs** and **ZIGIR** revealed Zn^2+^ dynamics within islets with high spatiotemporal resolution.^36–39^ Recently, our group reported a family of red- and far-red-emitting rhodamine-based Zn^2+^ probes called **PKZnR/FRs** with minimized phototoxicity, which enabled long-term 4D imaging of islet β-cell secretion due to their excellent biocompatibility and appropriate affinities for Zn^2+^.^40^ These excellent properties are derived from the hydrophilic morpholino auxochrome, which reduced the phototoxicity by hindering nonspecific binding and adjustable chelating groups. We also noticed that the morpholino auxochrome limited their brightness by leaving the saturated quantum yields of **PKZnRs** under 10%. We hypothesized that the limited quantum yield resulted from the “Twisted Intramolecular Charge Transfer (TICT)” effect of rhodamine-based fluorescent probes. The early solution to avoid the TICT state is to reduce the steric hindrance effect of auxochromes. Although this strategy can significantly enhance the brightness of fluorophores, it often leads to an increase in its hydrophobicity. In 2019 and 2020, Xiao and Guo groups reported a series of new auxochromes that contained strong electron withdrawing groups (quaternary piperazine or thiomorpholine 1,2-dioxide) to inhibit the TICT state.^41,42^ Inspired by these pioneering works, we assumed that replacing the morpholine moiety of the **PKZnRs** by a strong electron withdrawing group might significantly upgrade the brightness and hydrophilicity of probes for monitoring Zn^2+^/insulin corelease in islets.

In this study, we designed and synthesized a brand new class of PK Zinc Bright Red (**PKZnBR**) family with 4 candidates featuring the thiomorpholine-monoxide/1,1-dioxide auxochromes (Figure 1). Their quantum yields were generally increased ∼7 times over the old generation **PKZnR** probes. Better turn-on ratio (up to 134), appropriate binding affinity (10^–7^ to 10^–4^ M), and water solubility make them powerful tools for *ex vivo* recording of insulin secretion compared to existing Zn^2+^ probes (Figure S1).

**Figure 1:**
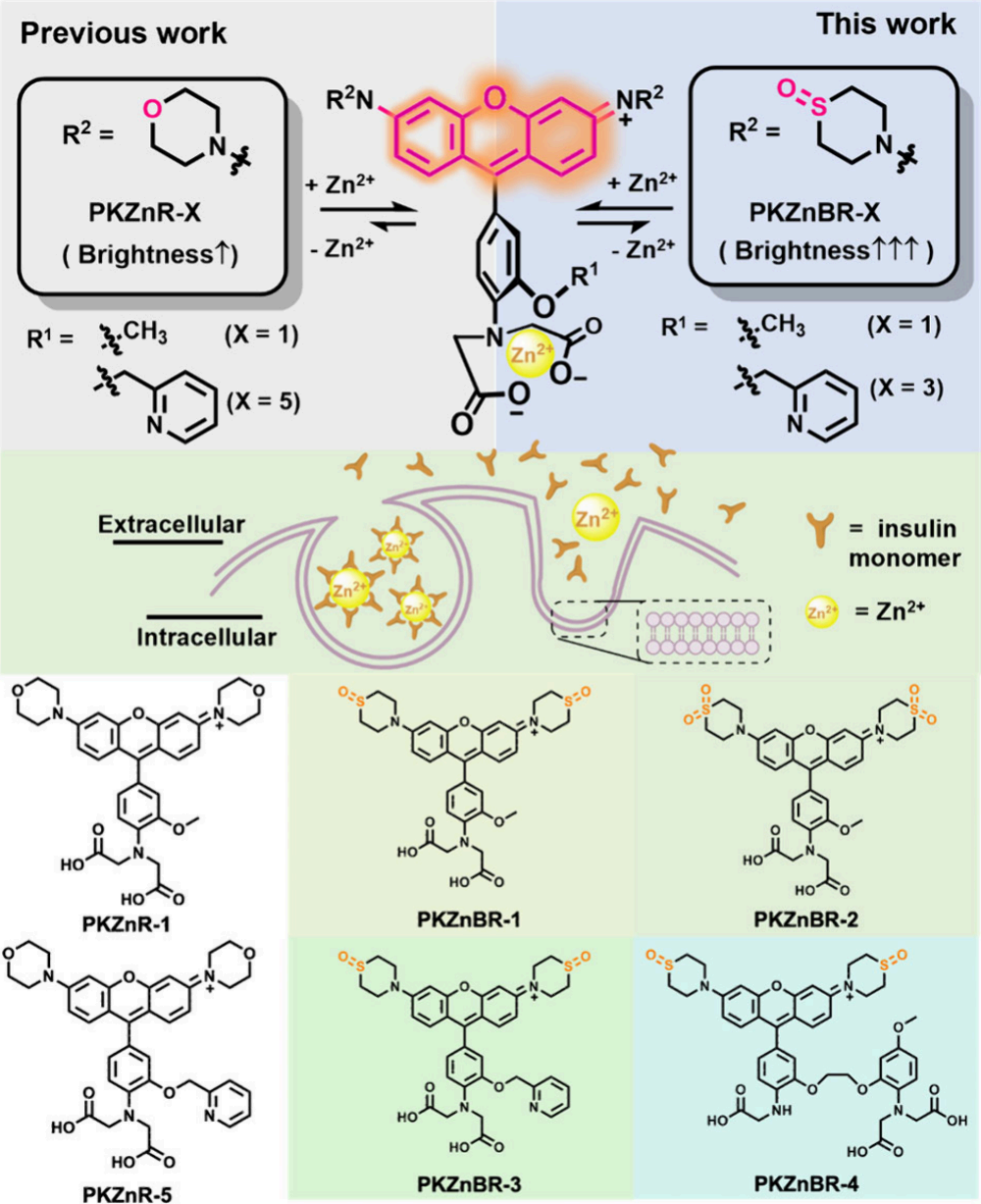
A new generation PK Zinc Bright Red dye (**PKZnBR**) featuring the thiomorpholine-monoxide auxochrome moiety exhibits brighter fluorescence signals in visualizing Zn^2+^/insulin corelease events.

## Experimental Section

### Tests of Photophysical Properties

Details of the photophysical tests are given in the Supporting Information.

### Synthesis of **PKZnBR-1–4**

The synthesis routes of **PKZnBR-1–4** are shown in Scheme 1. Experimental details are given in the Supporting Information. For the synthesis of Cpd 3, the commercially available 3-bromoanisole was fused with a thiomorpholine moiety through the Buchwald–Hartwig coupling reaction and oxidation. The aldehyde intermediates (Cpd 4–6) were synthesized according to the previous work, and the conditions of key Friedel–Crafts reactions between the aldehyde intermediates and Cpd 3 were generally identical. Finally, the ester protecting groups were removed by a saponification reaction to obtain the final products. Their characterization results are as follows.

**Scheme 1:**
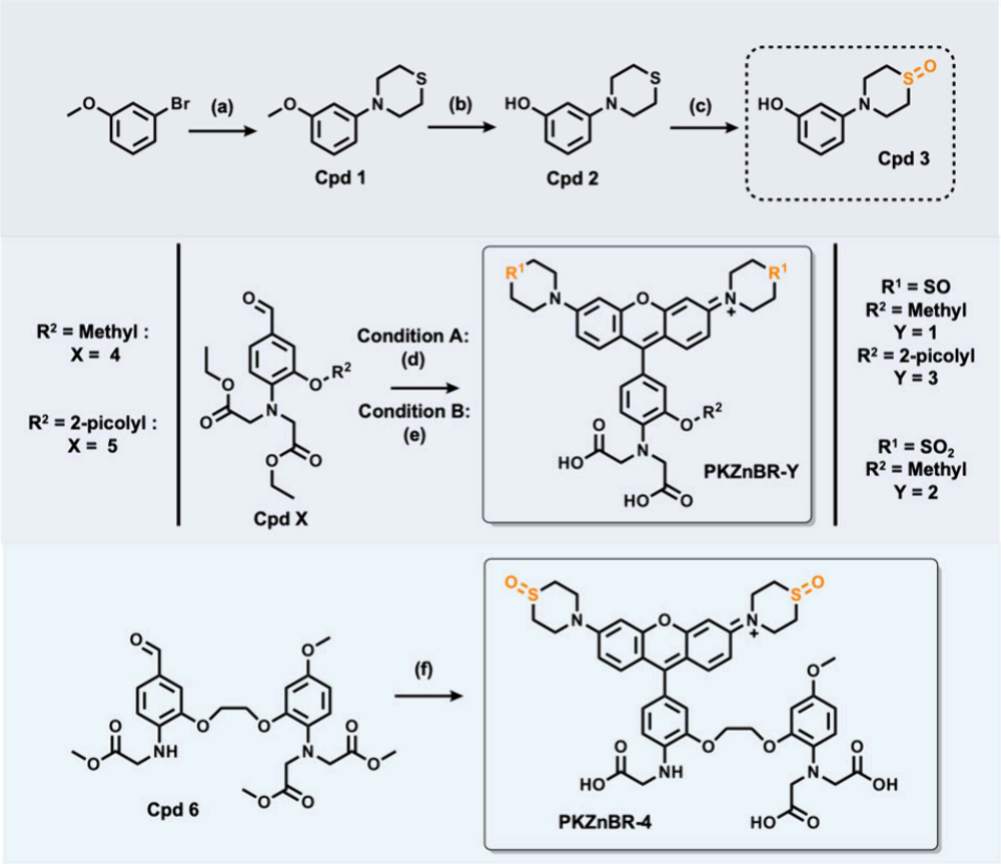
Syntheses of **PKZnBR-1–4** ^a^

a (a) Thiomorpholine, Pd­(OAc)_2_, BINAP, ^*t*^BuOK/toluene, 80 °C, 80% yield. (b) BBr_3_/DCM, 0 °C, 85% yield. (c) H_2_O_2_/HFIP, r.t., 50% yield. (d) (i) Cpd 3, Cpd 4 (yielding PKZnBR-1)/Cpd 5 (yielding PKZnBR-3), TsOH/TFA, 80 °C. (ii) DDQ, DCM/MeOH, r.t. (iii) LiOH, H_2_O/MeOH, r.t., 22% yield (PKZnBR-1), 15% yield (PKZnBR-3). (e) (i) Cpd 3, Cpd 4, TsOH/TFA, 80 °C. (ii) DDQ, DCM/MeOH, r.t. (iii) Sodium tungstate dihydrate, H_2_O_2_/ACN, r.t. (iv) LiOH, H_2_O/MeOH, r.t., 5% yield. (f) (i) Cpd 3, Cpd 6, Propanoic acid, 90 °C. (ii) Chloranil, MeOH/Chloroform, r.t. (iii) LiOH, H_2_O/MeOH, r.t., 3% yield.

#### PKZnBR-1

^1^H NMR (400 MHz, DMSO-*d*_6_) δ 7.61 (d, *J* = 9.6 Hz, 2H), 7.46 (dd, *J* = 9.7 Hz, 2.3 Hz, 2H), 7.34 (d, *J* = 2.3 Hz, 2H), 7.10 (d, *J* = 1.9 Hz, 1H), 7.04 (dd, *J* = 8.3 Hz, 1.9 Hz,1H), 6.87 (d, *J* = 8.3 Hz, 1H), 4.30 (d, *J* = 14.9 Hz, 4H), 4.18–4.12 (m, 8H), 3.75 (s, 3H), 3.07–3.00 (m, 4H), 2.88 (d, *J* = 13.6 Hz, 4H).

^13^C NMR (101 MHz, DMSO-*d*_6_) δ 172.1, 157.94, 157.89, 155.8, 149.4, 141.4, 132.5, 123.8, 122.3, 116.3, 115.2, 114.8, 113.5, 97.8, 56.1, 54.0, 44.6, 39.2.

HRMS­(ESI) calcd for C_32_H_34_N_3_O_8_S_2_^+^ [M]^+^ 652.1782, found 652.1785.

#### PKZnBR-2

^1^H NMR (400 MHz, DMSO-*d*_6_) δ 7.67 (d, *J* = 9.6 Hz, 2H), 7.50 (dd, *J* = 9.6 Hz, 2.2 Hz, 2H), 7.41 (d, *J* = 2.2 Hz, 2H), 7.12 (d, *J* = 1.8 Hz, 1H), 7.06 (dd, *J* = 8.3 Hz, 1.8 Hz, 1H), 6.88 (d, *J* = 8.3 Hz, 1H), 4.25 (bs, 8H), 4.17 (s, 4H), 3.76 (s, 3H), 3.34 (bs, 8H).

^13^C NMR (101 MHz, DMSO-*d*_6_) δ 172.1, 159.0, 157.9, 155.9, 149.3, 141.6, 132.6, 124.1, 122.1, 116.3, 115.7, 114.9, 114.0, 98.5, 56.1, 54.0, 51.0, 45.8.

HRMS­(ESI) calcd for C_32_H_34_N_3_O_10_S_2_^+^ [M]^+^ 684.1680, found 684.1677.

#### PKZnBR-3

^1^H NMR (400 MHz, DMF-*d*_7_) δ 8.60–8.59 (m, 1H), 7.93 (td, *J* = 7.7 Hz, 1.6 Hz, 1H), 7.67 (d, *J* = 7.9 Hz, 1H), 7.59 (d, *J* = 9.6 Hz, 2H), 7.50 (dd, *J* = 9.6 Hz, 2H), 7.45–7.42 (m, 3H), 7.30 (d, *J* = 1.7 Hz, 1H), 7.19 (dd, *J* = 8.4 Hz, 1.8 Hz, 1H), 7.13 (d, *J* = 8.3 Hz, 1H), 5.37 (s, 2H), 4.50 (s, 2H), 4.45 (bs, 6H), 4.36–4.29 (m, 4H), 3.26–3.20 (m, 4H), 2.98 (d, *J* = 13.6 Hz, 4H).

^13^C NMR (400 MHz, DMF-*d*_7_) δ 173.4, 163.4, 159.4, 157.8, 157.3, 150.4, 149.7, 143.3, 138.3, 133.6, 125.6, 124.2, 124.0, 123.1, 118.6, 117.7, 116.2, 114.9, 99.1, 72.6, 55.0, 46.2, 40.4.

HRMS­(ESI) calcd for C_37_H_37_N_4_O_8_S_2_^+^ [M]^+^ 729.2047, found 729.2042.

#### PKZnBR-4

^1^H NMR (600 MHz, DMSO-*d*_6_) δ 7.71 (d, *J* = 9.6 Hz, 2H), 7.41 (dd, *J* = 9.7 Hz, 2.2 Hz, 2H), 7.30 (d, *J* = 2.2 Hz, 2H), 7.14 (d, *J* = 1.5 Hz, 1H), 7.06 (dd, *J* = 8.1 Hz, 1.5 Hz, 2H), 6.78 (d, *J* = 8.9 Hz, 1H), 6.74 (d, *J* = 8.4 Hz, 1H), 6.60 (d, *J* = 2.9 Hz, 1H), 6.45 (dd, *J* = 8.7 Hz, 2.7 Hz, 1H), 4.39 (s, 4H), 4.26 (d, *J* = 14.6 Hz, 4H), 4.14 (t, *J* = 13.2 Hz, 4H), 4.02 (s, 2H), 3.96 (s, 4H), 3.68 (s, 3H), 3.05–3.01 (m, 4H), 2.88 (d, *J* = 13.3 Hz, 4H).

^13^C NMR (151 MHz, DMSO-*d*_6_) δ: 172.5, 171.9, 158.6, 157.8, 155.6, 154.9, 151.0, 145.0, 140.8, 132.8, 132.7, 125.4, 120.3, 118.2, 115.0, 113.5, 113.4, 109.1, 105.2, 101.9, 97.8, 67.14, 67.06, 55.3, 53.8, 44.6, 44.1, 40.0.

HRMS­(ESI)­calculated for C_42_H_45_N_4_O_12_S_2_^+^ [M]^+^ 861.2470, found 861.2475.

### Imaging of Glucose-Stimulated Exocytosis of Insulin Vesicles

To image insulin granules exocytosis, islets were cultured on a 35 mm glass bottom confocal dish (Cellvis, D35-14-1-N) for 24 h and then washed twice and bathed in prewarmed KRBB solution containing 125 mM NaCl, 5.9 mM KCl, 2.4 mM CaCl_2_, 1.2 mM MgCl_2_, 1 mM l-Glutamine, 25 mM HEPES, 3 mM glucose, 0.1% bovine serum albumin, and 10 μM Zn^2+^-probe for ∼15 min to silent the β-cells’ activity. Next, stimulated islets with a KRBB solution containing designated glucose and 10 μM Zn^2+^-dyes were imaged. All fluorescence images were acquired with a spinning-disc confocal microscope based on a CSU-X1 Yokogawa head mounted on an inverted IX-81 Olympus microscope. Images were acquired by a 60× (NA1.35, Olympus) oil immersion objective lens and at a sampling rate of ∼1 Hz. Finally, 10 μM FM4-64 (Invitrogen, T3166) was applied to label the plasma membrane of islet cells after islets were stimulated by glucose.

## Results and Discussion

### Probe Design

We choose thiomorpholine monoxide/thiomorpholine-1,1-dioxide to replace morpholino auxochromes in the fluorophore section and expected the following benefits: (1) They have strong electron-withdrawing effects that can prevent the formation of TICT states, increasing the brightness and turn-on ratio of probes. (2) They have extremely high water solubility that can minimize nonspecific staining of probes, allowing them to stay outside the cell membrane with minimal phototoxicity. (3) Two different electron-withdrawing auxochromes can fine-tune the spectrum and binding affinity of the probes. **PKZnBR-1** and **-2** feature 2-methoxyaniline-*N*,*N*-diacetate chelator while **PKZnBR-3** installs 2-pyridylmethyl on the side chain to obtain a higher affinity and more specific selectivity. We also introduced a BAPTA-like chelating group into **PKZnBR-4**, which had been reported to have more coordinating atoms and higher binding affinity.

### Characterization of **PKZnBR-1–4** *in Vitro*

With **PKZnBR-1–4** in hand, we tested their photophysical properties *in vitro* (Figures 2 and S2 to S5), including Zn^2+^ dependent change in absorption, fluorescence (Figure 2b), titration (Figure 2c), and selectivity against other divalent cations and biological active species. Compared to the previous **PKZnRs** probes, the corresponding **PKZnBR** probes displayed a blue shift on their absorption and emission spectra for 6 or 18 nm (Figure 2a), mainly because the sulfone group can reduce the electron-donating ability of the chromophores. Consistent with our expectations, the introduction of electron withdrawing auxochromes significantly improved the brightness of probes. To be specific, **PKZnBR-1** obtained a higher brightness than **PKZnBR-2** and **PKZnR-1** (45% vs 22% and 6%), and the turn-on ratio of **PKZnBR-1** (indicated by Δ*F*/*F*_0_) is also higher than the others­(50 vs 39 and 23), which indicates that the formation of the TICT state and probes’ photophysical properties do not precisely match a linear relationship on some occasions. It is worth noting that the introduction of thiomorpholine monoxide/thiomorpholine-1,1-dioxide also reduced the binding affinity of the probes. We hypothesized that it was attributed to the sulfur oxides’ electronic withdrawing effect, which might decrease the electronic density of the carboxyl group and nitrogen atom of the chelating group. Summarizing the above data, we found that probes containing thiomorpholine monoxide autochrome feature higher quantum yields, turn-on ratios, binding affinities, and red-shifted spectra than those of thiomorpholine-1,1-dioxide auxochrome. Thus, we applied this auxochrome in the design of **PKZnBR-3** and **-4**. **PKZnBR-3** is the brightest probe in the **PKZnBR** family, since its quantum yields can be 70%, which is 6.7 times higher than that of **PKZnR-5** with the same chelating group (Figure 2a). Furthermore, it is also a hydrophilic candidate, which will enormously reduce phototoxicity by minimizing nonspecific binding in tissue-level insulin-Zn^2+^ imaging. **PKZnBR-4** has the highest affinity within the **PKZnBR** family by following the design of **RhodZin-3**. Although a 160 nM-level *K*_d_ was accomplished, the afforded **PKZnBR-4** exhibited a lower saturated quantum yield than that of **PKZnBR-3**. Counting on their higher affinities, **PKZnBR-3** and **-4** should have the capability to detect Zn^2+^/insulin corelease in living systems with different secretion abilities.

**Figure 2:**
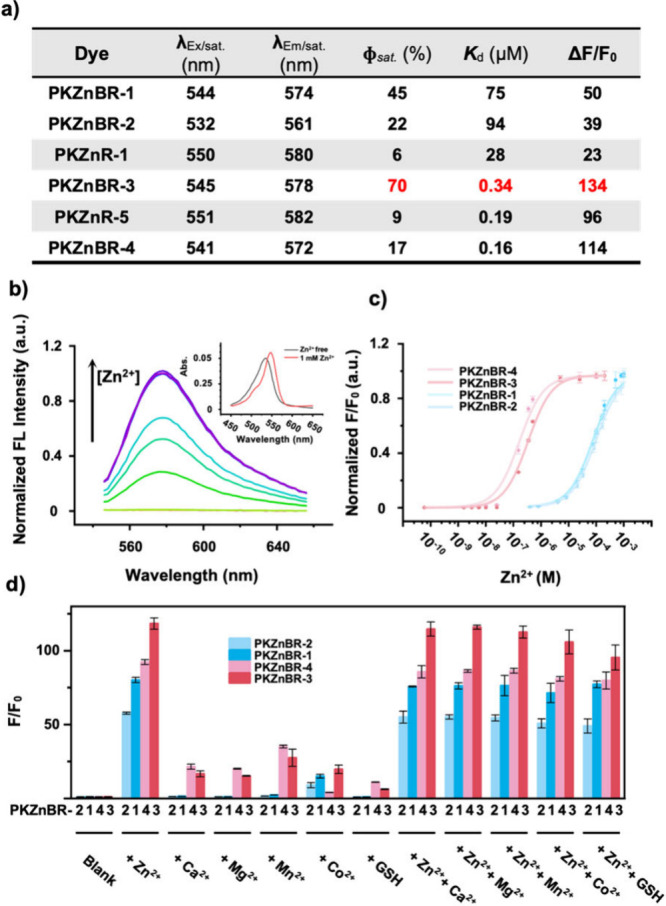
Characterizations of **PKZnBR-1–4** *in vitro*. (a) Photophysical properties of **PKZnBRs** (λ_Ex/sat._ and λ_Em/sat._: the maximum of excitation and emission wavelength when the probe was saturated by 1 mM Zn^2+^; ϕ_sat._: fluorescence quantum yield when the probe was saturated by 1 mM Zn^2+^; *K*_d_: dissociation constant). (b) Normalized fluorescence intensity of **PKZnBR-3** (1 μM) in buffers containing different concentrations of Zn^2+^. (c) Normalized **PKZnBRs’** (1 μM) binding curve fitted from individual emission maxima. For **PKZnBR-2**, a light blue color; for **PKZnBR-4**, a light red color; for **PKZnBR-1**, a deep blue color; for **PKZnBR-3**, a deep red color. (d) Selectivity and cross-talk trials. Zn^2+^, Ca^2+^, Mg^2+^, Mn^2+^, and Co^2+^ (1 mM) and GSH (10 mM) were added to **PKZnBRs** (1 μM). For **PKZnBR-2**, light blue column; for **PKZnBR-4**, light red column; for **PKZnBR-1**, deep blue column,; for **PKZnBR-3**, deep red column. Measurements were performed in HEPES buffer (100 mM HEPES, pH = 7.4, *I* (NaNO_3_) = 0.1, <0.5% DMSO as cosolvent), which contained different concentrations of Zn^2+^. For the concentration of free Zn^2+^ < 100 nM, 10 mM NTA was added to control the free Zn^2+^ concentration. 10 μM TPEN was added to ensure the free Zn^2+^ condition. Excitation/emission wavelength pairs of corresponding tests were set at 480/562 nm (for **PKZnBR-2**), 496/572 nm (for **PKZnBR-4**), 496/578 nm (for **PKZnBR-3**), and 495/573 nm (for **PKZnBR-1**) separately. Error bars denote SD; *n* = 3.

In the test of selectivity, **PKZnBR-1** and **PKZnBR-2** showed a negligible fluorescence response to the metal ions from the first and second main groups, which exactly matched what we had expected (Figure 2d). Furthermore, transition metal ions, such as Mn^2+^ and Co^2+^, were also trialed. The results indicated that Co^2+^ might slightly disturb Zn^2+^ recognition but Mn^2+^ almost did not. Besides, glutathione (GSH) was examined for roughly evaluating the interference resulting from biological electrophilic species, and it was proven to be ignorable in Zn^2+^ sensing events. **PKZnBR-3** and **PKZnBR-4** showed minor crosstalk effects on the listed metal ions and GSH, supposing an increase of coordination number might induce misrecognition chances. The above experiments demonstrate that the **PKZnBR** family has strong selectivity and robustness for Zn^2+^ against other metal ions or some important biologically active species.

Due to its superior brightness, turn-on ratio, and appropriate affinity, we selected **PKZnBR-3** for monitoring the corelease of insulin and Zn^2+^ in intact mouse islets.^40,43^ Upon stimulation with 18.2 mM glucose at 37 °C, we observed the abrupt emergence of dozens of brightly fluorescent puncta across the islet, indicative of physiologically relevant exocytosis of insulin granules (Figure 3a and Movie S1). The high hydrophilicity of **PKZnBR-3** enabled a nonphototoxic recording of Zn^2+^/insulin corelease for over 500 s, free from nonspecific staining (Figure 3a). The long-term recording revealed that, (1) within the intact islets, a significant increase in fluorescent puncta occurred approximately 1 min after glucose stimulation, lasting for about 5 min before gradually diminishing (Figure 3b). This biphasic pattern of insulin secretion by β-cells, with an initial robust phase followed by a subdued subsequent phase under high glucose stimulation,^40,43^ was clearly reflected by **PKZnBR-3**, demonstrating its reliability in capturing the dynamics of insulin secretion. (2) Analysis of individual fusion events identified three distinct modes of fusion in pancreatic islet β-cells.^40^ The first, termed “full fusion”, involves rapid and complete fusion of vesicles with the plasma membrane, primarily occurring within 1 s (ROI 2 in Figure 3c). The second mode, “short-lived fusion”, likely involves granules fusing with the cell membrane and gradually dissolving their insulin crystals (ROI 3 in Figure 3c). The third, “long-lived fusion”, might involve secretory vesicles fusing with the plasma membrane through a small pore to release their contents (ROI 4 in Figure 3c). Notably, the diameter of these fluorescent puncta ranged from 0.2 to 0.5 μm (Figure 3d). Furthermore, numerous diffuse signals were observed in the intercellular space as the stimulation continued. Given that the concentration of insulin-bound Zn^2+^ within β-cell vesicles can reach millimolar levels,^40,43^ the release of bound Zn^2+^ upon secretion to the extracellular environment increases extracellular Zn^2+^ concentration, resulting in these diffuse signals. These data confirm that PKZnBR-3, with its enhanced photophysical and chemical properties, is a potent sensor for *ex vivo* insulin recording.

**Figure 3:**
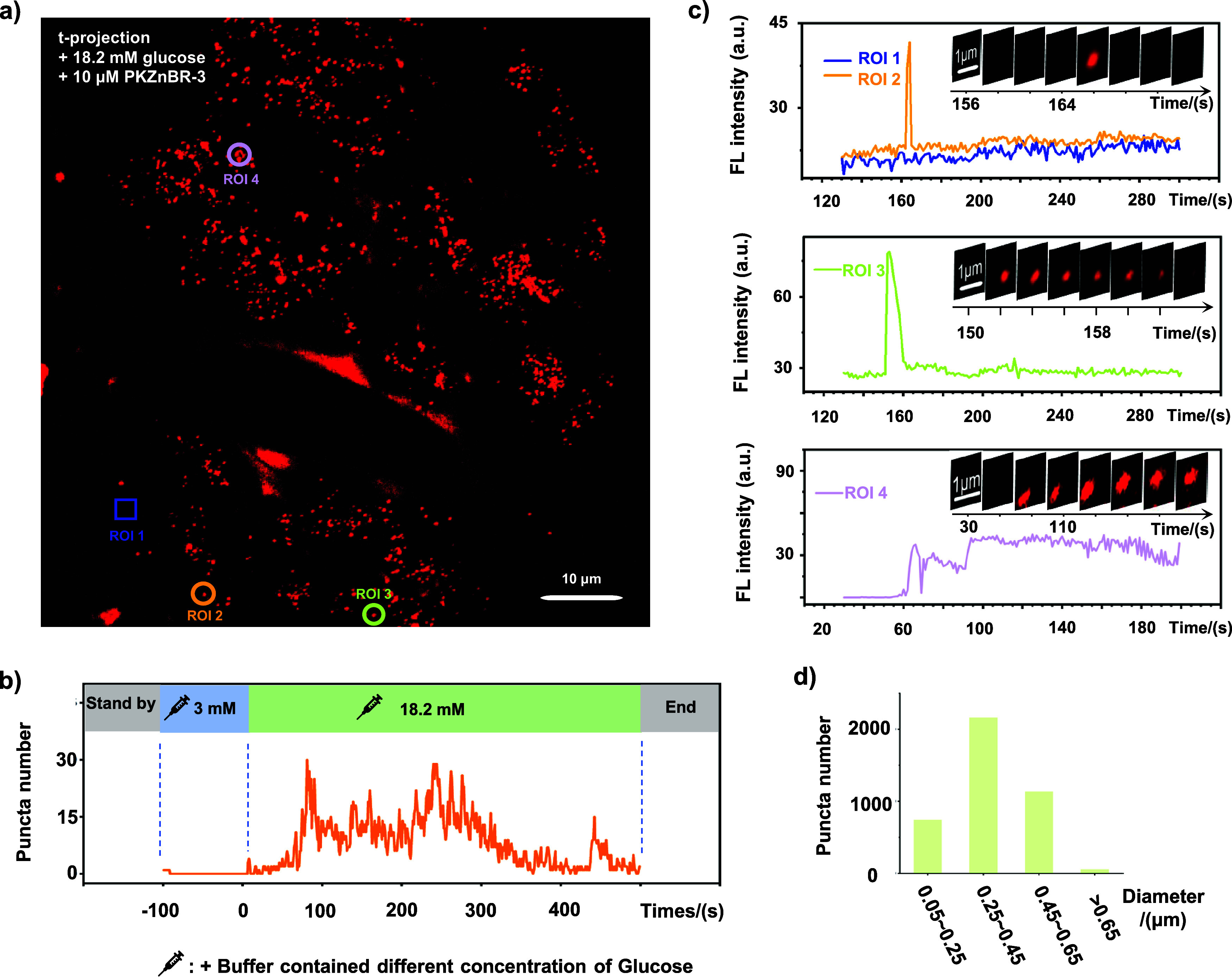
**PKZnBR-3**-powered visualization of three types of Zn^2+^/insulin fusion modes in living mouse islet. (a) The t-projection of fluorescence signals detected by **PKZnBR-3** under a high concentration of glucose stimulation (18.2 mM). Scale bar: 10 μm. ROI: Region of interest. (b) The number of Zn^2+^/insulin coreleasing puncta over time during continued stimulation. Puncta was extracted from ImageJ through a built-in particle analysis function. (c) The fluorescence intensity curves over time within selected regions of interest (ROIs) (the inset montages show consecutive image series of corresponding ROIs at 2 s (ROI 1–3) or 10 s (ROI 4)/frame. Scale bar = 1 μm). (d) Distribution of diameters of fluorescent dots.

## Conclusions

In summary, we introduced thiomorpholine monoxide auxochrome into Zn^2+^ probes and obtained a brand new class of Zn^2+^ probes (**PKZnBR**) featuring higher brightness (∼7×), better turn-on ratio (up to 134), and improved hydrophilicity than the previous version (**PKZnR**). From a chemical perspective, this work indicated the significant advantages of introducing modern auxochrome engineering strategies into the design of ion probes. From a biological perspective, our work expanded the tool kit of extracellular Zn^2+^ probes for long-term monitoring of insulin secretion with minimal phototoxicity, highlighting the potential impact of biocompatible sensors on β-cell endocrinology.

## Supplementary Material





## Notes

General information, experimental details, map summarizing the state-of-the-art Zn^2+^ probes with different excitation/emission wavelengths and binding affinities for biological studies, characterization data, and NMR data (PDF)
PKZnBR-3 reveals three types of Zn2+/insulin fusion modes in living mouse islet (AVI)
