# Supplementary material for: Bright Zinc Probes with Thiomorpholine Monoxide Auxochromes for Imaging Insulin Secretion
Source: Chem Biomed Imaging. 2025 Mar 4;3(7):455–61. doi: 10.1021/cbmi.4c00116 (PMC12308593; doi:10.1021/cbmi.4c00116)
Supplement: Supplementary file 1 [file im4c00116_si_001.pdf]

Supporting Information for

Bright Zinc Probes with Thiomorpholine Monoxide Auxochromes for Imaging Insulin Secretion

Rundong Yu[a,b§], Junwei Zhang[a§], Xiaohong Peng[c,d*], Zhixing Chen[a,b*]

[a]State Key Laboratory of Membrane Biology, Institute of Molecular Medicine, National Biomedical Imaging Center, Beijing Key Laboratory of Cardiometabolic Molecular Medicine, College of Future Technology, Peking University; Beijing 100871, China.
[b]Peking-Tsinghua Center for Life Sciences, Academy for Advanced Interdisciplinary Studies, Peking University; Beijing 100871, China.
[c]School of Basic Medical Sciences, Shenzhen University Medical School, Shenzhen 518060, China.
[d]Department of Physiology and Pathophysiology, School of Basic Medical Sciences, Peking University, Beijing 100191, China.
§These authors contributed equally to this work.
*Corresponding authors: zhixingchen@pku.edu.cn; xhpeng@szu.edu.cn.

Table of Content

Supporting Figure S1-5 -- 2

General Experimental Information of Imaging and *in vitro* Tests--------------- 7

Chemical Synthesis and Characterization of New Compounds ---------------- 9

NMR Spectra -- 18

References -- 29

Supporting Figure S1 – 5

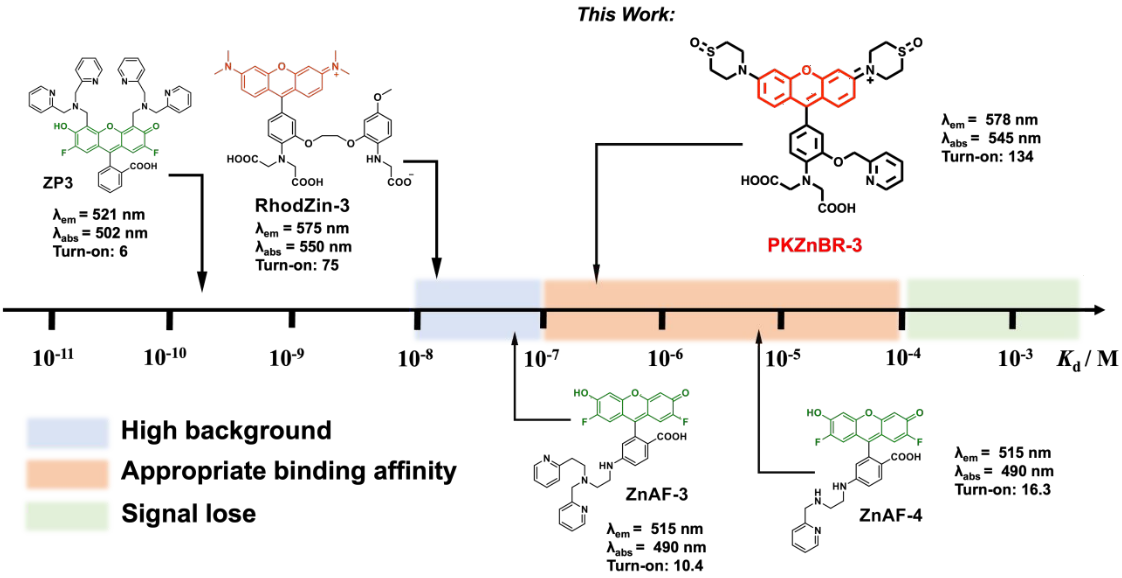

Figure S1. A map summarizing the state-of-the-art Zn^{2+} probes with different excitation/emission wavelengths and binding affinities for biological studies.

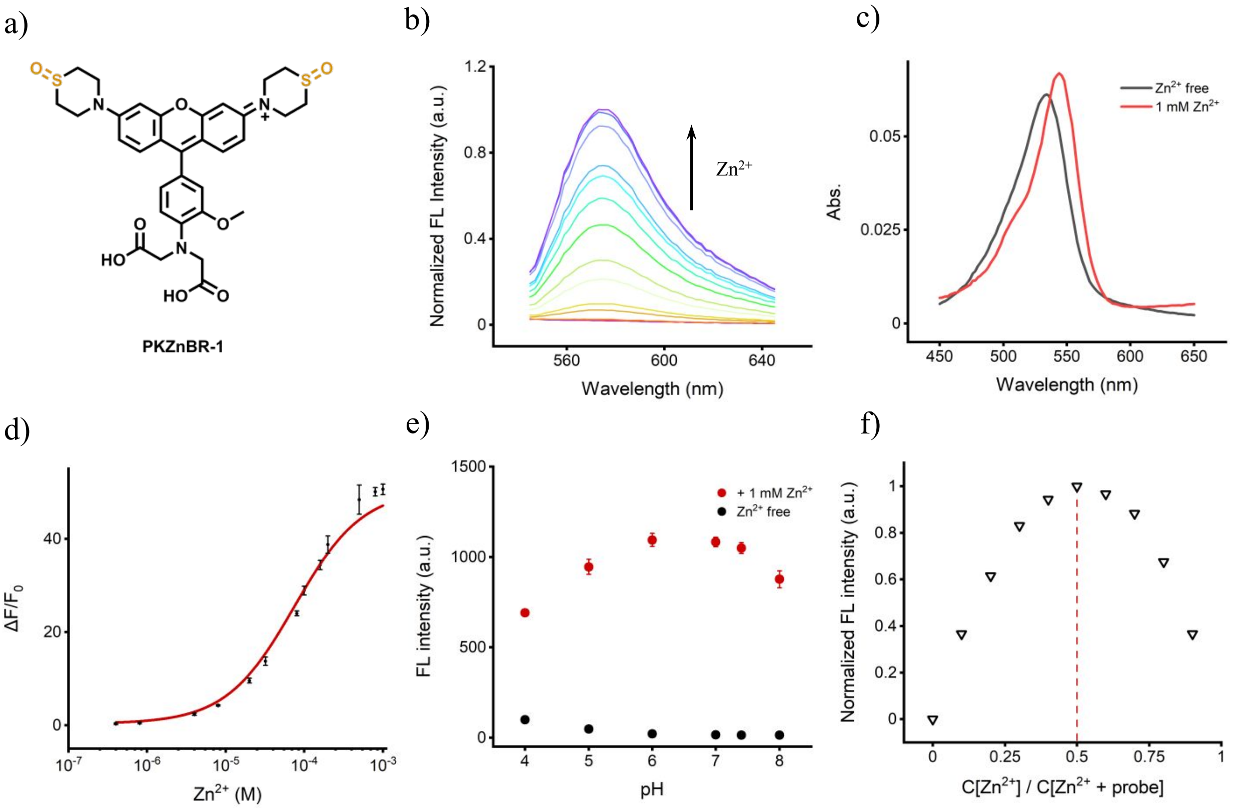

g)

Dye	Zn^{2+} free		Zn^{2+} saturated bound			K_d (μM)	ΔF/F_0
	λ_{Ex} (nm)	λ_{Em} (nm)	λ_{Ex} (nm)	λ_{Em} (nm)	Φ_{sat} (%)		
PKZnBR-1	534	569	544	574	45	75	50

Figure S2. Characterizations of PKZnBR-1. (a). The chemical structure of **PKZnBR-1**. (b) Emission spectra of **PKZnBR-1** (1 μM) in the presence of various concentrations of free Zn^{2+} (0, 0.4, 0.8, 4, 8, 20, 32, 80, 100, 160, 200, 500, 800, 1000 μM). (c) Absorption spectra of **PKZnBR-1** (1 μM) in the presence of 0 and 1 mM free Zn^{2+}. (d). Zn^{2+} titration of **PKZnBR-1** (1 μM) as measured from its emission at 574 nm. (e) pH dependence of fluorescence for **PKZnBR-1** in 0 and 1 mM free Zn^{2+}. (f) Job's plot of **PKZnBR-1**. The sum of the concentration of Zn^{2+} and **PKZnBR-1** is 10 μM. (g) Photophysical properties of **PKZnBR-1**. Measurements were performed in HEPES buffer (100 mM HEPES, pH = 7.4, *I* ($NaNO_3$) = 0.1, <0.5 % DMSO as co-solvent). Zero Zn^{2+} measurements were made in the presence of 10 μM TPEN. The excitation wavelength was 494 nm. Error bars denote SD; *n* = 3.

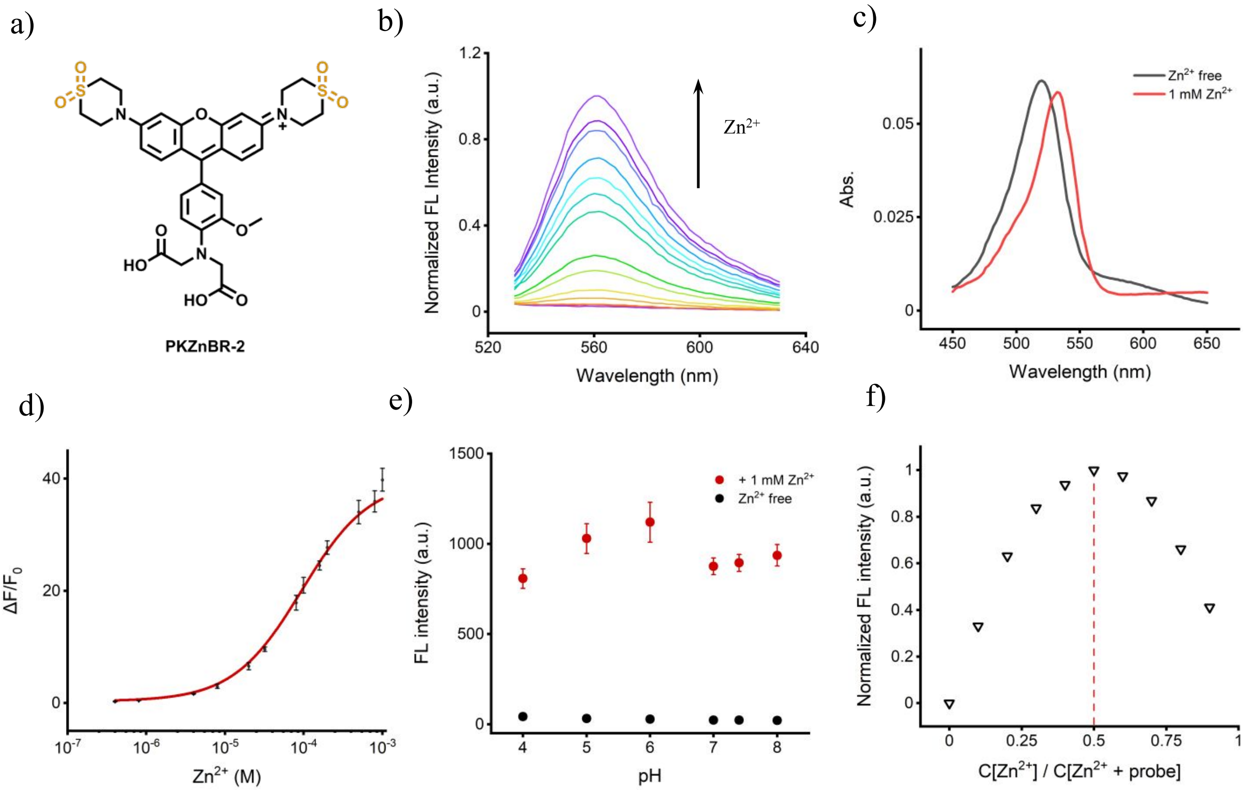

g)

Dye	Zn^{2+} free		Zn^{2+} saturated bound			K_d (μM)	ΔF/F$_0$
	λ_{Ex} (nm)	λ_{Em} (nm)	λ_{Ex} (nm)	λ_{Em} (nm)	ϕ_{sat} (%)		
PKZnBR-2	520	556	532	561	22	94	39

Figure S3. Characterizations of PKZnBR-2. (a). The chemical structure of **PKZnBR-2**. (b) Emission spectra of **PKZnBR-2** (1 μM) in the presence of various concentrations of free Zn^{2+} (0, 0.4, 0.8, 4, 8, 20, 32, 80, 100, 160, 200, 500, 800, 1000 μM). (c) Absorption spectra of **PKZnBR-2** (1 μM) in the presence of 0 and 1 mM free Zn^{2+}. (d). Zn^{2+} titration of **PKZnBR-2** (1 μM) as measured from its emission at 562 nm. (e) pH dependence of fluorescence for **PKZnBR-2** in 0 and 1 mM free Zn^{2+}. (f) Job's plot of **PKZnBR-2**. The sum of the concentration of Zn^{2+} and **PKZnBR-2** is 10 μM. (g) Photophysical properties of **PKZnBR-2**. Measurements were performed in HEPES buffer (100 mM HEPES, pH = 7.4, I ($NaNO_3$) = 0.1, <0.5 % DMSO as co-solvent). Zero Zn^{2+} measurements were made in the presence of 10 μM TPEN. The excitation wavelength was 480 nm. Error bars denote SD; n = 3.

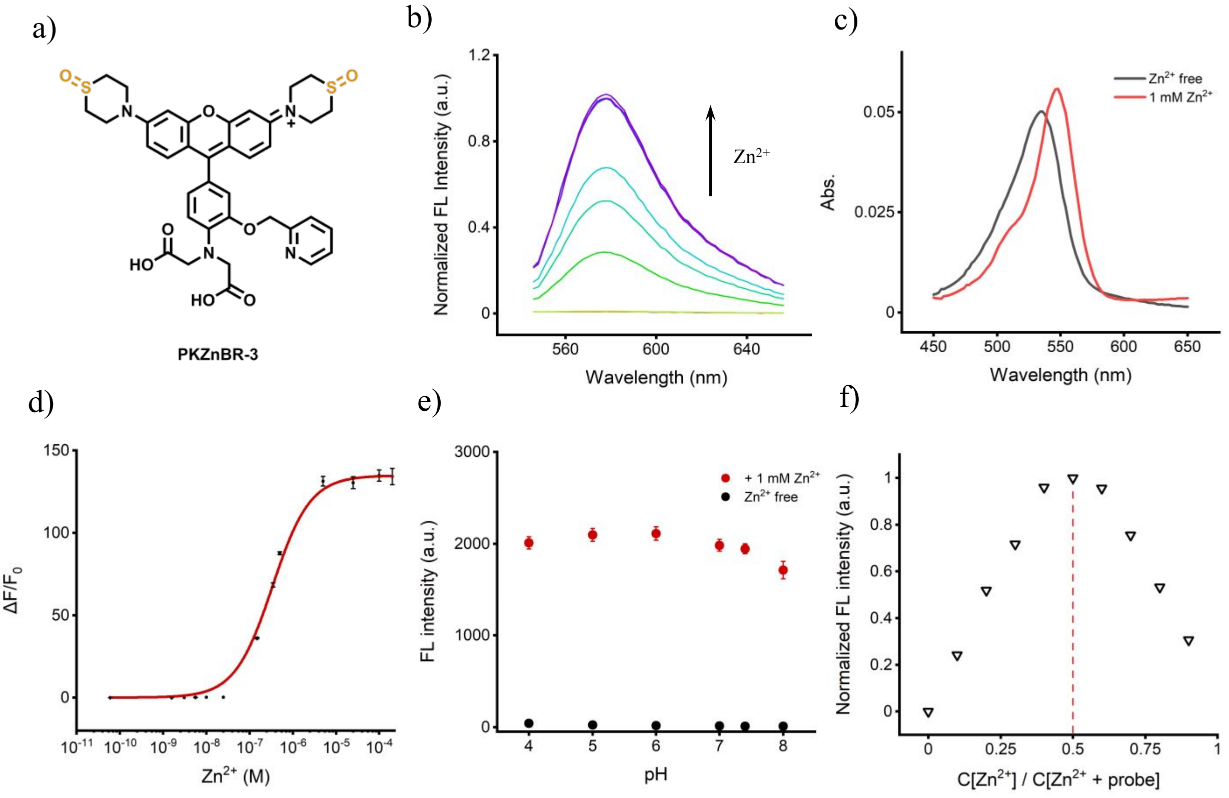

g)

Dye	Zn^{2+} free		Zn^{2+} saturated bound			K_d (μM)	ΔF/F_0
	λ_{Ex} (nm)	λ_{Em} (nm)	λ_{Ex} (nm)	λ_{Em} (nm)	Φ_{sat} (%)		
PKZnBR-3	535	573	545	578	70	0.34	134

Figure S4. Characterizations of PKZnBR-3. (a). The chemical structure of **PKZnBR-3**. (b) Emission spectra of **PKZnBR-3** (1 μM) in the presence of various concentrations of free Zn^{2+} (0, 0.00006, 0.0016, 0.0031, 0.0056, 0.01, 0.025, 0.15, 0.35, 0.5, 5, 25, 100, 200 μM). (c) Absorption spectra of **PKZnBR-3** (1 μM) in the presence of 0 and 1 mM free Zn^{2+}. (d). Zn^{2+} titration of **PKZnBR-3** (1 μM) as measured from its emission at 578 nm. (e) pH dependence of fluorescence for **PKZnBR-3** in 0 and 1 mM free Zn^{2+}. (f) Job's plot of **PKZnBR-3**. The sum of the concentration of Zn^{2+} and **PKZnBR-3** is 10 μM. (g) Photophysical properties of **PKZnBR-3**. Measurements were performed in HEPES buffer (100 mM HEPES, pH = 7.4, I ($NaNO_3$) = 0.1, <0.5 % DMSO as co-solvent). Zero Zn^{2+} measurements were made in the presence of 10 μM TPEN. The excitation wavelength was 495 nm. Error bars denote SD; n = 3.

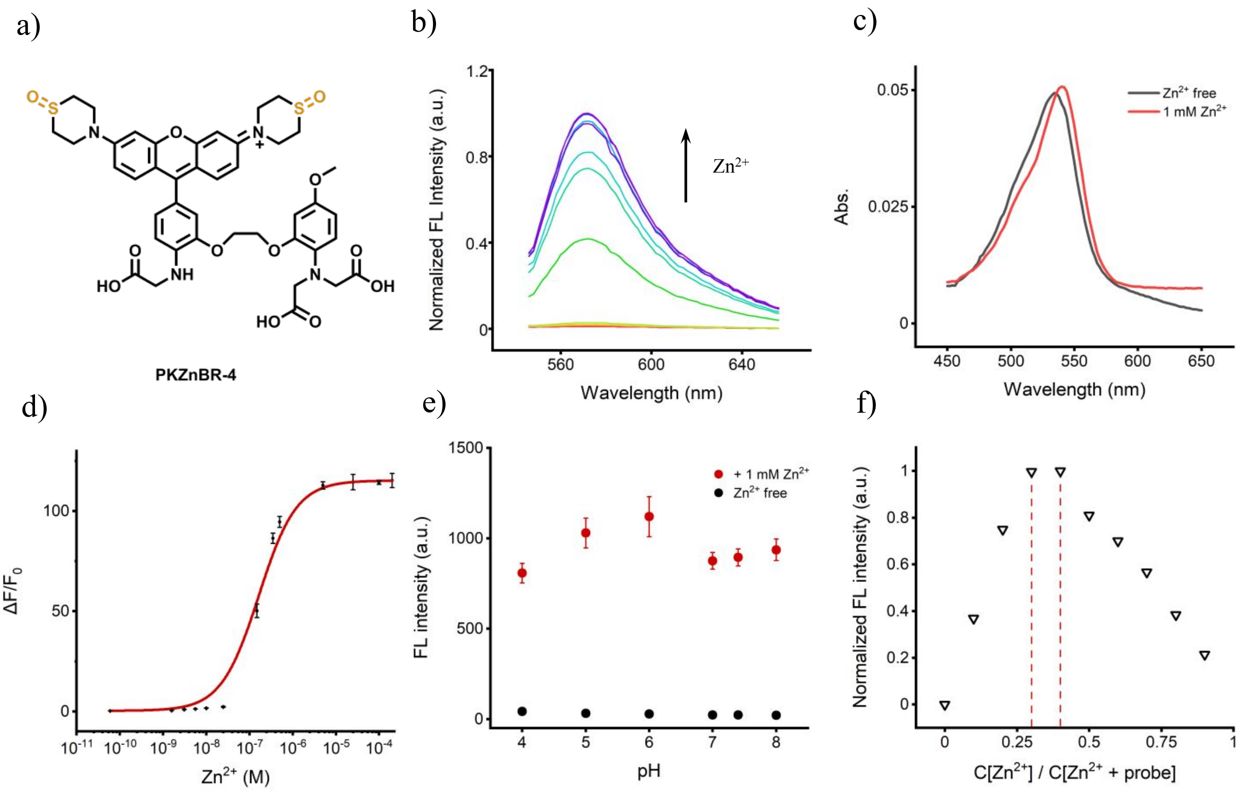

g)

Dye	Zn^{2+} free		Zn^{2+} saturated bound			K_d (μM)	ΔF/F$_0$
	λ_{Ex} (nm)	λ_{Em} (nm)	λ_{Ex} (nm)	λ_{Em} (nm)	Φ_{sat} (%)		
PKZnBR-4	535	568	541	572	17	0.16	114

Figure S5. Characterizations of PKZnBR-4. (a). The chemical structure of **PKZnBR-4**. (b) Emission spectra of **PKZnBR-4** (1 μM) in the presence of various concentrations of free Zn^{2+} (0, 0.00006, 0.0016, 0.0031, 0.0056, 0.01, 0.025, 0.15, 0.35, 0.5, 5, 25, 100, 200 μM). (c) Absorption spectra of **PKZnBR-4** (1 μM) in the presence of 0 and 1 mM free Zn^{2+}. (d). Zn^{2+} titration of **PKZnBR-4** (1 μM) as measured from its emission at 572 nm. (e) pH dependence of fluorescence for **PKZnBR-4** in 0 and 1 mM free Zn^{2+}. (f) Job's plot of **PKZnBR-4**. The sum of the concentration of Zn^{2+} and **PKZnBR-4** is 10 μM. (g) Photophysical properties of **PKZnBR-4**. Measurements were performed in HEPES buffer (100 mM HEPES, pH = 7.4, I ($NaNO_3$) = 0.1, <0.5 % DMSO as co-solvent). Zero Zn^{2+} measurements were made in the presence of 10 μM TPEN. The excitation wavelength was 495 nm. Error bars denote SD; n = 3.

General Experimental Information of Imaging and *in vitro* Tests

Absorbance and fluorescence spectrum

All photophysical measurements, including UV-vis absorption spectra and emission spectra, were gained on a microplate reader (TECAN Infinite M Nano+, Switzerland) in 96-well plates at ambient conditions (30 ± 2 °C). All **PKZnBR** dyes were stocked in DMSO and frozen in -20 °C refrigerator. Upon being prepared for tests, the DMSO stock solutions were diluted by water to a 1/10 concentration of the stock solutions. After that, in order to keep the quota of co-solvent DMSO under 0.5% v/v, appropriate volume of the diluted dye solution was mixed with the HEPES buffer pre-charged into the units of a 96-well plate by pipettes to arrive at a final concentration of **PKZnBR**s around 1 μM.

Dissociation constant measurement

The fluorescence intensity of **PKZnBR**s (1 μM) was collected under the conditions containing various concentrations of $ZnSO_4$. The conditions containing over 100 nM free Zn^{2+} was prepared by directly diluting high concentration Zn^{2+} with HEPES buffer (100 mM HEPES, pH = 7.4 , *I* ($NaNO_3$) = 0.1). The conditions containing under 100 nM free Zn^{2+} was prepared according to the previous work. Zn^{2+} free conditions were assured by adding 10 μM TPEN to the HEPES buffer.

And the dissociation constants (K_d) of Zn^{2+} were gained by fitting the following equation:

$$F = (F_{min}K_d + F_{max}[Zn^{2+}])/(K_d + [Zn^{2+}])$$

Selectivity tests

A serious of materials stock solutions were prepared as 20 mM aqueous solutions, which contained $ZnSO_4$, $CaSO_4$, $MgSO_4$, $MnSO_4$, $CoSO_4$, GSH separately. Upon measuring, appropriate volume of the stock solutions were injected into HEPES buffer (100 mM HEPES, pH = 7.4, *I* ($NaNO_3$) = 0.1) followed by the addition of **PKZnBR**s DMSO/H_2O solution, to form a final concentration of 1 mM metal ions and 1 μM **PKZnBR**s in the units of a 96-well plate. The crosstalk effect was detected by combining $ZnSO_4$ stock solution and another stock solution in the same units of a 96-well plate and assuring the two final concentrations equals to 1 mM. Free Zn^{2+} conditions were made by adding 10 μM TPEN towards the HEPES buffer.

Quantum yields determination

PKZnBRs' quantum yields were identified by comparing the brightness with Rhodamine B (relative determination method). The maximum fluorescence response curve was afforded by measuring the fluorescence intensity with the conditions containing 1 mM Zn^{2+} and the minimum fluorescence response curve was afforded by measuring the intensity under Zn^{2+} conditions, which was assured by 10 μM TPEN existing in HEPES buffer. Absorbance intensity at corresponding excitation wavelength did not beyond 0.1 for both PKZnBRs and reference. The Zn^{2+}-saturated quantum yields (Φ_{sat}) of the PKZnBRs and Zn^{2+}-free quantum yields (Φ_{free}) of the PKZnBRs were calculated according to the following equation (the reported Φ_{sat} and Φ_{free} were mean values of 4 tests):

$$\Phi_{sample} / \Phi_{ref} = A_{ref} I_{sample} d^2_{sample} / A_{sample} I_{ref} d^2_{ref}$$

Φ: fluorescence quantum yield;

I: integrated value of the emission intensity;
A: Absorbance intensity at corresponding excitation wavelength;
d: refractive index of solvents, d_{water} = 1.33; d_{MeOH} = 1.329.

Islet isolation and culture

Ins1-GCaMP6f mice were crossbred by the Ins1-Cre (Jackson Laboratories, stock number 026801) and GCaMP6f $^{fl/fl}$ lines (Jackson Laboratories, stock number 029626). Primary islets were isolated by collagenase p digestion and then purified by hand-picking. Harvested islets were allowed to recover overnight in RPMI 1640 supplemented with 10% FBS, 8 mM glucose, and 100 U/mL and 100 mg/mL Pen/Strep. All procedures of animal breeding and experimental manipulations were approved by the Peking University Animal Use and Care Committee and complied with the standards of the Association for Assessment and Accreditation of Laboratory Animal Care.

Imaging of glucose-stimulated insulin vesicles

To imaging insulin granules exocytosis, islets were cultured on a 35 mm glass bottom confocal dish (Cellvis, D35-14-1-N) for 24 hours, and then washed twice and bathed in pre-warmed KRBB solution containing 125 mM NaCl, 5.9 mM KCl, 2.4 mM $CaCl_2$, 1.2 mM $MgCl_2$, 1 mM L-Glutamine, 25 mM HEPES, 3 mM glucose, 0.1% bovine serum albumin, and 10 μM Zn^{2+}-probe for ~15 minutes to silent β-cells' activity. Next, stimulated islets with KRBB solution containing designated glucose and 10 μM Zn^{2+}-dyes were imaged. All fluorescence images were acquired with a spinning-disc confocal microscope based on a CSU-X1 Yokogawa head mounted on an inverted IX-81 Olympus microscope. Images were acquired by a 60X (NA1.35, Olympus) oil immersion objective lens and at a sampling rate of ~1 Hz. Finally, 10 μM **FM4-64** (Invitrogen, T3166) was applied to label the plasma membrane of islet cells after islets were stimulated by glucose.

Image analysis

Treating the living islet to **PKZnBR** dyes and different levels of glucose results in fluorescence signals, which was collected by confocal microscope as high-resolution images. They are packed together sequentially and analyzed by ImageJ. Typically, the overview of images' stack were adjusted to be fine by manually changing the threshold and picking a color from the look up table. The t-projection of the images' stack was formed for a general view of Zn^{2+}-insulin co-release signals. By trapping special regions with colored cycles or frames, the dynamic information of individual signals was extracted and shown in Figure 3c. The cards queue inserted in the graphs of Figure 3c were partially copied from the corresponding ROIs and exhibited the detailed life of the corresponding signals.

The temporal distribution of releasing signals and diameters of detected signals were analyzed by ImageJ and Origin. By using the built-in functions of ImageJ, the images' stack was transferred into a binary form followed by filtering noise to avoid extra or mistake counting. Abstract signals' number and size could be directly readout from practicles analysis function of ImageJ. Such information was transferred into graphs by using Origin.

Chemical Synthesis and Characterization of New Compounds.

General Information

All reactions were contained in dry sealed flasks and carried out with rotating stir bars under ambient conditions, unless otherwise noted. Reactions performed in high temperature were heated in oil bath. Solvents mentioned in procedures were commercially available and applied into reactions without further purification. To be specific, toluene (99.5 %, AR), dichloromethane (DCM, 99.9 %，AR), methanol (MeOH, 99.9 %, CCER) and chloroform (99 %, AR) were purchased from TGREAG (Beijing, China) hexafluoroisopropanol (HFIP, 98 %), trifluoroacetic acid (TFA, 99.5 %), propanoic acid (99 %), were purchased from Energy Chemical (Beijing, China), acetonitrile (ACN, 99.9 %, CCER) were purchased from Concord Technology (China). H_2O refers to refined water (conductivity < 1 μS/cm). Unless otherwise stated, other chemicals involved in reactions were either purchased with highest quality and directly charged in reactions without any purification or synthesized according to previous work.

Thin layer chromatography (TLC) silica gel plates were supplied by Yantai Chemicals (China) and used to monitor reaction procedural with a portable TLC visualizer UV 254/365. Further visualization of TLC was completed by the participation of phosphomolybdic acid, iodine or $KMnO_4$-stain. Silica gel (300-400 mesh), purchased from Tsingtao Haiyang Chemicals (China), was used to perform flash column chromatography.

PKZnBRs and their esterified forms were purified by high performance liquid chromatography (HPLC) performed on Teledyne Isco EZ Prep UV-Vis equipped with a RediSep Prep C18 column (100 Å, 5 μm, 20 x 150 mm). The afforded aqueous solutions were frozen to proper form and removed the solvents by a lyophilizer (SONG YUAN FREEZE DRYER, China, LGJ-12 equipped with a Welch CRVpro8 Vacuum Pump). Primary identification of target molecule was supported by analytic LC/MS (4.6 mm × 150 mm 5 μm C18 column; 2 μL injection; 5–100% CH_3CN/H_2O, linear gradient, with constant 0.1 % v/v formic acid additive; 6-10 min run; 0.4 mL/min flow; ESI; positive or negative ion mode; UV detection with ACQUITY PDA).

NMR spectra were recorded on Bruker Avance series equipment (^{1}H 400 MHz, ^{13}C 101 MHz or ^{1}H 600 MHz, ^{13}C 151 MHz) and were calibrated using residual undeuterated solvent (Chloroform-*d* at 7.26 ppm ^{1}H NMR, 77.16 ppm ^{13}C NMR; DMSO-d_6 at 2.50 ppm ^{1}H NMR, 39.52 ppm ^{13}C NMR; DMF-d_7 at 8.03 ppm ^{1}H NMR, 163.44 ppm ^{13}C NMR). Data for ^{1}H NMR spectra are arranged as follows: chemical shift (δ ppm), multiplicity (s = singlet, d = doublet, t = triplet, q = quartet, dd = doublet of doublets, dt = triplet of doublets, m = multiplet, bs = broad singlet), coupling constant (Hz), integration. Data for ^{13}C NMR are reported by chemical shift (δ ppm). High-resolution mass spectrometric data were collected from Fourier Transform Ion Cyclotron Resonance Mass Spectrometer, Solarix XR, Bruker, using ESI (electrospray ionization).

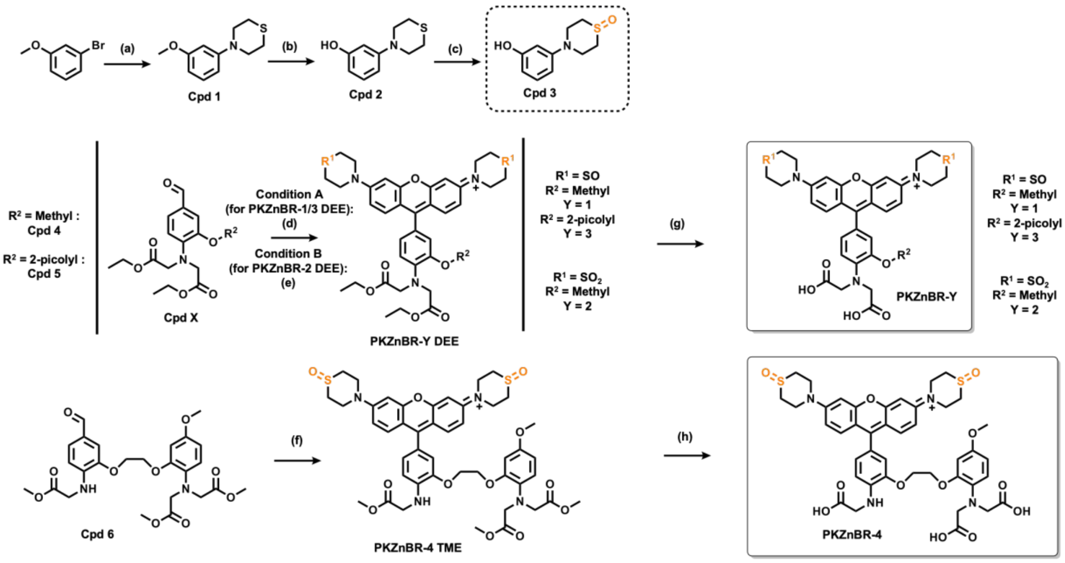

Scheme S1. synthesis of **PKZnBR 1–4**: a) Thiomorpholine, $Pd(OAc)_2$, BINAP, ^{t}BuOK/toluene, 100°C, 80% yield. b) BBr_3/DCM, 0°C, 85% yield. c) H_2O_2/HFIP, r.t., 50% yield. d) i) Cpd 3, Cpd 4 (yielding PKZnBR-1)/ Cpd 5 (yielding PKZnBR-3), TsOH/TFA, 80°C ii) DDQ, DCM/MeOH, r.t.. e) i) Cpd 3, Cpd 4, TsOH/TFA, 80°C ii) DDQ, DCM/MeOH, r.t. iii) Sodium tungstate dihydrate, H_2O_2/ACN, r.t.. g) LiOH, H_2O/MeOH, r.t., 5% yield (for PKZnBR-2 over 4 steps), 22% yield (for PKZnBR-1 over 3 steps), 15% Yield (for PKZnBR-3 over 3 steps). f) i) Cpd 3, Cpd 6, propanoic acid, 90°C ii) Chloranil, MeOH/chloroform, r.t.. h) LiOH, H_2O/MeOH, r.t., 3% yield.

Detailed process and identification of key compounds

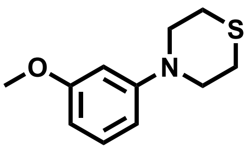

Cpd 1 To a dry 100 mL multi-necked flask was added a Teflon-coated stir bar, $Pd(OAc)_2$ (83 mg, 0.50 mmol), ^{t}BuONa (1.4 g, 15 mmol), BINAP (620 mg, 1.0 mmol), then the flask was equipped with a condenser and recharged by nitrogen gas for 3 times through a simplified Schlenk line. After that, 3-bromoanisole (1.9 g, 10 mmol) and thiomorpholine (1.5 g, 15 mmol), which were dissolved in 20 mL dry toluene, were injected into the flask. then the flask was heated for 3 hours in an oil bath at 100°C under nitrogen atmosphere. After cooling down, the mixture was filtered through a Celite pad to yield deep brown liquid which was concentrated under reduced pressure and then purified by silica gel chromatography. The eluent was a linear gradient combination of petroleum ether and ethyl acetate from 80/1 to 40/1(v/v). These efforts finally afforded a light-yellow oil with 80% yield (1.7 g).

^{1}HNMR (400 MHz, $CDCl_3$) □ 7.17 (t, J = 8.1 Hz, 1H), 6.51 (d, J = 8.1 Hz, 1H), 6.42 (d, J = 8.4 Hz, 2H), 3.79 (s, 3H), 3.57 ~ 3.54 (m, 4H), 2.75 ~ 2.72 (m, 4H).

^{13}CNMR (101 MHz, $CDCl_3$) □ 160.8, 152.6, 130.1, 109.8, 104.5, 103.6.

HRMS (ESI) calculated for $C_{11}H_{16}NOS^+[M+H]^+$ 210.0947, found 210.0946.

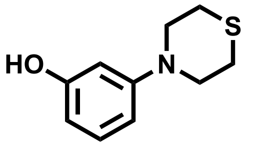

Cop 2 A dry 50 mL multi-neck flask was equipped with a Teflon-coated stir bar. Then the inside atmosphere was replaced by nitrogen gas and 25 mL anhydrous DCM was added. Afterwards the flask was sealed and cooled in a crushed ice bath, then boron tribromide (9.0 mL, 93 mmol) and 2.0 mL anhydrous DCM dissolved **Cpd 1** (3.9 g, 19 mmol) were add to the flask slowly, removed the ice bath and rendered the flask warmed to room temperature for 3 hours. Then the mixture is carefully poured into cold methanol and subsequently concentrated under reduced pressure, the residual was purified by silica gel column chromatography (In order to yield pure product, the purification steps should be handled in a nitrogen charger glove box to prevent the crude product from being oxidized to dark material) with a linear gradient eluent formed by petroleum ether and ethyl acetate from 10/1 to 3/1(v/v). Steps above offered products as white powder with 85 % yield (3.0 g).

^{1}HNMR (400 MHz, $CDCl_3$) □ 7.10 (t, J = 8.1 Hz, 1H), 6.47 (ddd , J = 8.4 Hz, 2.4 Hz, 0.6 Hz, 1H), 6.35 (t, J = 2.4 Hz, 1H), 6.31 (ddd, J = 8.4 Hz, 2.4 Hz, 0.7 Hz, 1H), 3.54 ~ 3.51 (m, 4H), 2.73 ~ 2.70 (m, 4H).

^{13}CNMR (101 MHz, $CDCl_3$) □ 156.7, 152.6, 130.4, 109.6, 106.8, 104.1, 52.0, 26.5.

HRMS (ESI) calculated for $C_{10}H_{14}NOS^+$ $[M+H]^+$ 196.0791, found 196.0789.

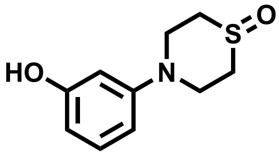

Cpd 3 A flask was equipped with a Teflon-coated stir bar and **Cpd 2** (800 mg 4.1 mmol) dissolved in 10 mL HFIP to form a clear solution. Then the flask was surrounded by crushed ice to be cooled, followed by the dropwise injection of a ready-mixed mixture of 30% H_2O_2 (510 μL, 5.1 mmol) and 5 mL HFIP. Upon finishing injection, removed the ice bath and reacted in ambient conditions for one hour. Afterwards the solvent was removed under reduced pressure and the residue was transferred to a silica gel purification system (In order to yield pure product, the purification steps should be handled in a nitrogen charger glove box to prevent the crude product from being oxidized to dark solid), the eluent was set as a gradient combination of DCM and methanol from 15/1 to 7/1(v/v). Pure product was gained as white powder with 50 % yield (430 mg).

^{1}HNMR (400 MHz, DMSO-d_6) □ 9.18 (s, 1H), 7.01 (t, 8.1 Hz, 1H), 6.43 (dd, J = 8.1 Hz, 2.3 Hz, 1H), 6.37 (t, J = 2.3 Hz, 1H), 6.22 (dd, J = 8.0 Hz, 1.9 Hz, 1H), 3.76 ~ 3.69 (m, 2H), 3.54 ~ 3.49 (m, 2H), 2.93 ~ 2.86 (m, 2H), 2.67 ~ 2.62 (m, 2H).

^{13}CNMR (101 MHz, DMSO-d_6) □ 158.4, 150.3, 129.9, 106.6, 106.2, 102.6, 43.5, 39.9.

HRMS (ESI) calculated for $C_{10}H_{14}NO_2S^+$ $[M+H]^+$ 212.0740, found 212.0738.

General procedure A

A pressure tube was charged with a stir bar, corresponding aldehyde compound (0.10 mmol), **Cpd 3** (46 mg, 0.22 mmol), TsOH (3.5 mg, 0.020 mmol) and 1 mL TFA. Then the tube was sealed by a screw Teflon plug and heated overnight at 85°C. Upon Cooling down, the mixture was quenched by 50 mL cold saturated sodium acetate solution, and the obtained liquid was transferred to a separatory funnel, following the extraction with 100 mL chloroform for 3 times. Then combined the organic phase and dried over anhydrous Na_2SO_4, and then filtered the salts and evaporated the solvents under reduced pressure. The residual was dissolved with 1 mL DCM and 1 mL methanol to formed a clear solution which was subsequentially cooled by crushed ice and vigorously stirred until finishing the addition of DDQ (5.8 mg per portion, 3 portions in total, 0.076 mmol). Then removed ice bath and reacted at ambient conditions for 3 hours, resulted in a dark purple solvent which should be concentrated under cold vacuum. The residue was firstly purified by a silica gel column chromatography with an eluent formed by DCM /methanol (8/1, v/v). The afforded crude product was then refined by HPLC (eluent conditions: changing the quota of solvent B from 30% to 90% along a 35-min linear gradient; detection wavelength, 550 nm; eluent A (ddH_2O containing 0.1% TFA (v/v)) and eluent B (acetonitrile) ; flow rate, 8.0 mL/min) provided the **PKZnBR-Y DEE** (Diethyl Ester) solution which was subsequentially frozen to proper form and concentrated to dryness as trifluoroacetic salt (dark purple powder) under high vacuum.

General procedure B

The **PKZnBR** dye ester form (0.022 mmol, synthesized in **General procedure A**) was charged to a 20 mL vial, together with 4 mL methanol. Then prepared LiOH aqueous solution (4.0 mL 0.050 M) in another 20 mL vial and stirred the solution in a crushed ice bath. Upon cooling down, the intermediate compound/methanol was added to the LiOH solution dropwise followed by removing of the ice bath. The reaction was allowed to run at ambient conditions for 30 minutes, until another portion of LiOH aqueous solution (2.0 mL, 0.12 M) was add dropwise in a crushed ice bath. Then another 30 minutes was granted to the reaction under ambient conditions, until the last portion of LiOH solution (1.0 mL, 0.28 M) was add dropwise in a crushed ice bath. Steps above afforded a clear orange solution which was subsequentially concentrated under reduced pressure, and the residual was mixed with 1 mL cold DMF. Then the crude mixture was refined by HPLC (eluent conditions: changing the quota of solvent B from 20% to 90% along a 35-min linear gradient; detection wavelength, 550 nm; eluent A (ddH_2O containing 0.1% TFA (v/v)) and eluent B (acetonitrile) ; flow rate, 8.0 mL/min). The afforded solution was concentrated under reduced pressure and frozen to proper form which was subsequentially concentrated to dryness under vacuum. Pure product was gained as dark purple powder.

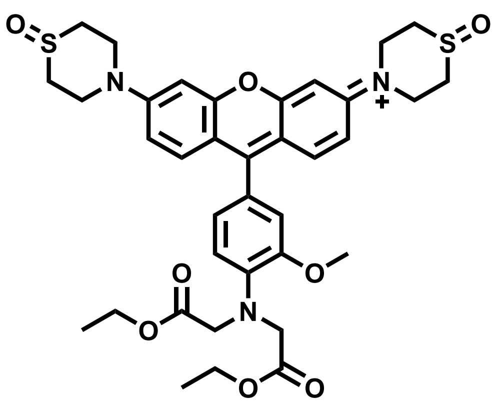

PKZnBR-1 DEE (PKZnBR-1 Diethyl Ester) This compound was synthesized according to **General procedure A** and the “corresponding aldehyde compound” mentioned in it referred to **Cpd 4** in this part.[1] The product was identified by NMR and HRMS and subsequentially treated by the following conditions.

^{1}HNMR (400 MHz, DMSO-d_6) □ 7.58 (d, J = 9.6 Hz, 2H), 7.45 (dd, J = 9.7 Hz, 2.3 Hz, 2H), 7.33 (d, J = 2.2 Hz, 2H), 7.10 (d, J =1.8 Hz, 1H), 7.02 (dd, J = 8.3 Hz, 1.8 Hz, 1H), 6.88 (d, J = 8.3 Hz, 1H), 4.30 (d, J = 14.9 Hz, 4H), 4.23 (s, 4H), 4.19 ~ 4.12 (m, 8H), 3.74 (s, 3H), 3.07 ~ 3.01 (m, 4H), 2.88 (d, J =13.7 Hz, 4H), 1.24 (t, J = 7.1 Hz, 6H).

^{13}CNMR (101 MHz, DMSO-d_6) □ 170.6, 157.9, 155.8, 149.5, 141.1, 132.4, 123.6, 122.7, 116.6, 115.2, 114.6, 114.0, 113.6, 97.7, 60.3, 56.1, 53.9, 44.6, 39.2, 14.2.

HRMS (ESI) calculated for $C_{36}H_{42}N_3O_8S_2^+$ $[M]^+$ 708.2408, found 708.2412.

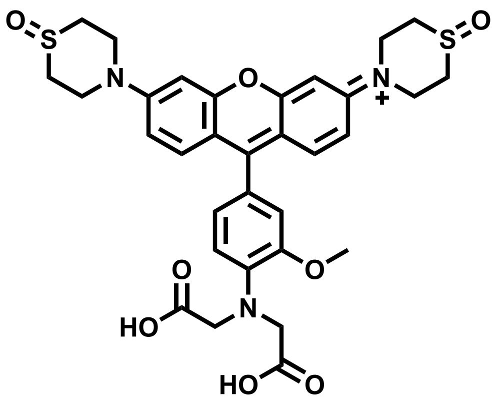

PKZnBR-1 This compound was synthesized according to **General procedure B** and the “PKZnBR dye ester form” mentioned in it referred to **PKZnBR-1 DEE** in this part. Yield: 22 % over 3 steps (14 mg).

^{1}HNMR (400 MHz, DMSO-d_6) □ 7.61 (d, J = 9.6 Hz, 2H), 7.46 (dd, J = 9.7 Hz, 2.3 Hz, 2H), 7.34 (d, J = 2.3 Hz, 2H), 7.10 (d, J =1.9 Hz, 1H), 7.04 (dd, J = 8.3 Hz, 1.9 Hz,1H), 6.87 (d, J = 8.3 Hz, 1H), 4.30 (d, J =14.9 Hz, 4H), 4.18 ~ 4.12 (m, 8H), 3.75 (s, 3H), 3.07 ~ 3.00 (m, 4H), 2.88 (d, J =13.6 Hz, 4H).

^{13}CNMR (101 MHz, DMSO-d_6) □ 172.1, 157.94, 157.89, 155.8, 149.4, 141.4, 132.5, 123.8, 122.3, 116.3, 115.2, 114.8, 113.5, 97.8, 56.1, 54.0, 44.6, 39.2.

HRMS (ESI) calculated for $C_{32}H_{34}N_3O_8S_2^+$ $[M]^+$ 652.1782, found 652.1785.

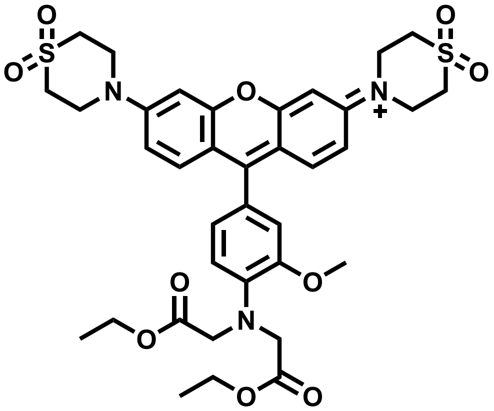

PKZnBR-2 DEE (PKZnBR-2 Diethyl Ester) The product (**PKZnBR-1 DEE**, 0.030 mmol) obtained from the **General procedure A** was charged in a 20 mL vial, together with a stir bar, 10 mL acetonitrile and sodium tungstate dihydrate (10 mg, 0.030 mmol). Then the vial was cooled in a crushed ice bath for 10 minutes, until 30% H_2O_2 (100 μL, 1.2 mmol) was slowly added to the mixture. Upon finishing the addition, the ice bath was removed and it was left to react under ambient conditions for 30 minutes. And then the vial was filtered through a 0.22 μm microfiltration membrane to afforded a deep red solution which was later diluted with 6 mL cold H_2O/DMF 1/1(v/v) mixture and concentrated under vacuum. The obtained solution was refined by HPLC (eluent conditions: changing the quota of solvent B from 25% to 90% along a 35-min linear gradient; detection wavelength, 550 nm; eluent A (ddH_2O containing 0.1% TFA (v/v)) and eluent B (acetonitrile) ; flow rate, 8.0 mL/min) provided the intermediate compound as trifluoroacetic salt (dark purple solid). The product was identified by NMR and HRMS and subsequentially treated by the following conditions.

^{1}HNMR (400 MHz, DMSO-*d*$_6$) □ 7.64 (d, *J* = 9.6 Hz, 2H), 7.49 (dd, *J* = 9.7 Hz, 2.0 Hz, 2H), 7.41 (d, *J* = 2.1 Hz, 2H), 7.12 (d, *J* = 1.7 Hz, 1H), 7.05 (dd, *J* = 8.4 Hz, 1.7 Hz, 1H), 6.89 (d, *J* = 8.3 Hz, 1H), 4.23 (bs, 12H), 4.17 (q, *J* = 7.1 Hz, 4H), 3.74 (s, 3H), 3.34 (bs, 8H), 1.24 (t, *J* = 7.1 Hz, 6H).

^{13}CNMR (101 MHz, DMSO-*d*$_6$) □ 170.6, 159.0, 158.0, 156.0, 149.5, 141.3, 132.6, 123.9, 122.6, 116.6, 115.7, 114.8, 114.0, 98.5, 60.3, 56.1, 54.0, 51.0, 45.8, 14.2.

HRMS (ESI) calculated for $C_{36}H_{42}N_3O_{10}S_2^+$ $[M]^+$ 740.2306, found 740.2301.

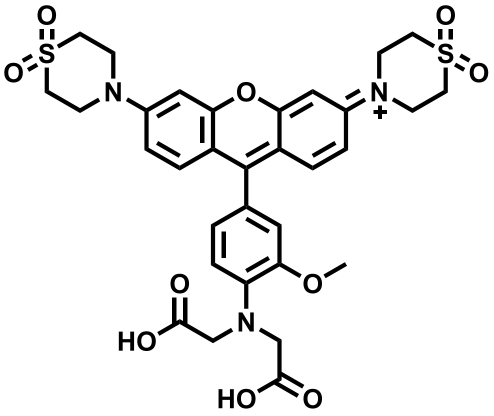

PKZnBR-2 This compound was synthesized according to **General procedure B** and the "PKZnBR dye ester form" mentioned in it referred to **PKZnBR-2 DEE** in this part. Yield: 5 % over 4 steps (3.4 mg).

^{1}HNMR (400 MHz, DMSO-d_6) □ 7.67 (d, J = 9.6 Hz, 2H), 7.50 (dd, J = 9.6 Hz, 2.2 Hz, 2H), 7.41 (d, J = 2.2 Hz, 2H), 7.12 (d, J = 1.8 Hz, 1H), 7.06 (dd, J = 8.3 Hz, 1.8 Hz, 1H), 6.88 (d, J = 8.3 Hz, 1H), 4.25 (bs, 8H), 4.17 (s, 4H), 3.76 (s, 3H), 3.34 (bs, 8H).

^{13}CNMR (101 MHz, DMSO-d_6) □ 172.1, 159.0, 157.9, 155.9, 149.3, 141.6, 132.6, 124.1, 122.1, 116.3, 115.7, 114.9, 114.0, 98.5, 56.1, 54.0, 51.0, 45.8.

HRMS (ESI) calculated for $C_{32}H_{34}N_3O_{10}S_2^+$ $[M]^+$ 684.1680, found 684.1677.

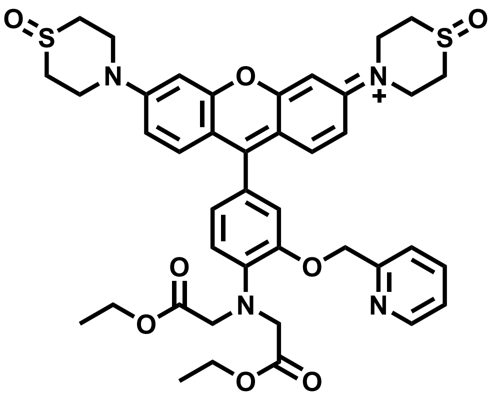

PKZnBR-3 DEE (PKZnBR-3 Diethyl Ester) This compound was synthesized according to **General procedure A** and the "corresponding aldehyde compound" mentioned in it referred to **Cpd 5** in this part.[1, 2] The product was identified by NMR and HRMS and subsequentially treated by the following conditions.

^{1}HNMR (400 MHz, DMSO-d_6) □ 8.56 (d, J = 4.8 Hz, 1H), 7.90 (td, J = 7.7 Hz, 1.7 Hz, 1H), 7.50 (d, J = 7.9 Hz, 1H), 7.44 (d, J = 9.6 Hz, 2H), 7.41 ~ 7.39 (m, 1H), 7.36 (dd, J = 9.7 Hz, 2.1 Hz, 2H), 7.32 (d, J = 2.1 Hz, 2H), 7.12 (d, J = 1.7 Hz, 1H), 7.04 (dd, J = 8.3 Hz, 1.7 Hz, 1H), 6.94 (d, J = 8.3 Hz, 1H), 5.21 (s, 2H), 4.31 (s, 6H), 4.28 (s, 4H), 4.15 (t, J = 12.9 Hz, 4H), 4.07 (q, J = 7.1 Hz, 4H), 3.07 ~ 3.00 (m, 4H), 2.89 (d, J = 13.9 Hz, 4H), 1.17 (t, J = 7.1 Hz, 6H).

^{13}CNMR (151 MHz, DMSO-d_6) □ 170.5, 157.9, 157.6, 156.0, 155.8, 149.1, 148.2, 141.3, 137.3, 132.2, 124.0, 123.2, 122.7, 121.9, 117.4, 115.9, 115.1, 113.4, 97.8, 71.1, 60.3, 53.7, 44.6, 39.2, 14.1.

HRMS (ESI) calculated for $C_{41}H_{45}N_4O_8S_2^+$ $[M]^+$ 785.2673, found 785.2670.

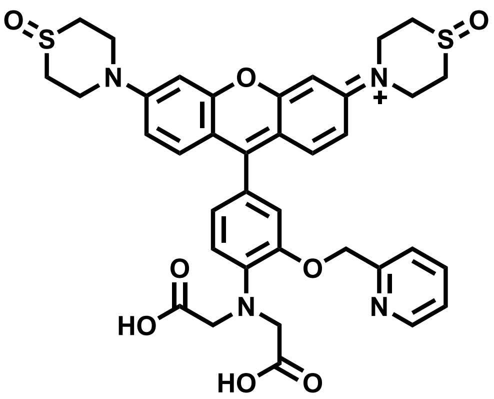

PKZnBR-3 This compound was synthesized according to **General procedure B** and the "PKZnBR dye ester form" mentioned in it referred to **PKZnBR-3 DEE** in this part. Yield: 15% over 3 steps (11 mg).
^{1}HNMR (400 MHz, DMF-d_7) □ 8.60 ~ 8.59 (m, 1H), 7.93 (td, J = 7.7 Hz, 1.6 Hz, 1H), 7.67 (d, J = 7.9 Hz, 1H), 7.59 (d, J = 9.6 Hz, 2H), 7.50 (dd, J = 9.6 Hz, 2H), 7.45 ~ 7.42 (m, 3H), 7.30 (d, J = 1.7 Hz, 1H), 7.19 (dd, J = 8.4 Hz, 1.8 Hz, 1H), 7.13 (d, J = 8.3 Hz, 1H), 5.37 (s, 2H), 4.50 (s, 2H), 4.45 (bs, 6H), 4.36 ~ 4.29 (m, 4H), 3.26 ~ 3.20 (m, 4H), 2.98 (d, J = 13.6 Hz, 4H).
^{13}CNMR (400 MHz, DMF-d_7) □ 173.4, 163.4, 159.4, 157.8, 157.3, 150.4, 149.7, 143.3, 138.3, 133.6, 125.6, 124.2, 124.0, 123.1, 118.6, 117.7, 116.2, 114.9, 99.1, 72.6, 55.0, 46.2, 40.4.
HRMS (ESI) calculated for $C_{37}H_{37}N_4O_8S_2^+$ $[M]^+$ 729.2047, found 729.2042.

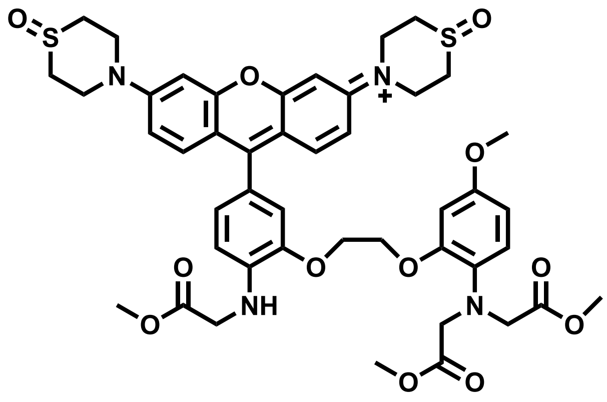

PKZnBR-4 TME (PKZnBR-4 Trimethyl Ester) A pressure tube was charged with a stir bar, **Cpd 6** (52 mg, 0.10 mmol), **Cpd 3** (46 mg, 0.22 mmol) and 0.5 mL propanoic acid.[1, 3, 4] Then the tube was sealed by a screw Teflon plug and heated for 30 hours at 90°C. Upon cooling down, the mixture was diluted by 15 mL chloroform, and the obtained liquid was transferred to a separatory funnel, following the washing with 60 mL saturated NaCl solution for 3 times. Then combined the organic phase and dried over anhydrous Na_2SO_4, and then filtered the salts. The afforded solvent was further mixed with 15 mL methanol, following by the addition of chloranil (27 mg, 0.11 mmol). Then left the mixture react at ambient conditions overnight, resulted in a dark purple solvent which should be diluted with 45 mL chloroform and washed through saturated NaCl solution for 3 times. This solvent was subsequentially dried through anhydrous Na_2SO_4 before loading on a silica gel column chromatography and the following purification by the eluent formed by DCM/methanol (8/1, v/v). the afforded crude product was then refined by HPLC (eluent conditions: changing the quota of solvent B from 30% to 90% along a 35-min linear gradient; detection wavelength, 550 nm; eluent

A (ddH_2O containing 0.1% TFA (v/v)) and eluent B (acetonitrile); flow rate, 8.0 mL/min) provided the intermediate compound as trifluoroacetic salt (dark purple solid). The product was identified by NMR and HRMS and subsequentially treated by the following conditions.

^{1}HNMR (600 MHz, DMSO-d_6) □ 7.69 (d, J = 9.6 Hz, 2H), 7.42 (dd, J = 9.7 Hz, 2.3 Hz, 2H), 7.31 (s, 2H), 7.13 (d, J =1.5 Hz, 1H), 7.04 (dd, J = 8.2 Hz, 1.4 Hz, 1H), 6.76 (t, J = 8.2 Hz, 2H), 6.59 (d, J = 2.6 Hz, 1H), 6.43 (dd, J = 8.7 Hz, 2.6 Hz, 1H), 4.36 (s, 4H), 4.28 (d, J = 15 Hz, 4H), 4.17 ~ 4.12 (m, 6H), 4.03 (s, 4H), 3.69 (s, 3H), 3.68 (s, 3H), 3.51 (s, 6H), 3.05 ~ 3.01 (m, 4H), 2.88 (d, J =13.3 Hz, 4H).

^{13}CNMR (151 MHz, DMSO-d_6) □ 171.4, 171.1, 158.6, 157.8, 155.6, 155.0, 151.0, 145.1, 140.6, 132.7, 132.3, 125.3, 119.9, 118.4, 115.0, 113.4, 113.3, 109.1, 104.8, 101.4, 97.8, 67.1, 66.8, 55.3, 53.3, 51.8, 51.2, 44.6, 44.0, 40.0.

HRMS (ESI) calculated for $C_{45}H_{51}N_4O_{12}S_2^+$ $[M]^+$ 903.2939, found 903.2933.

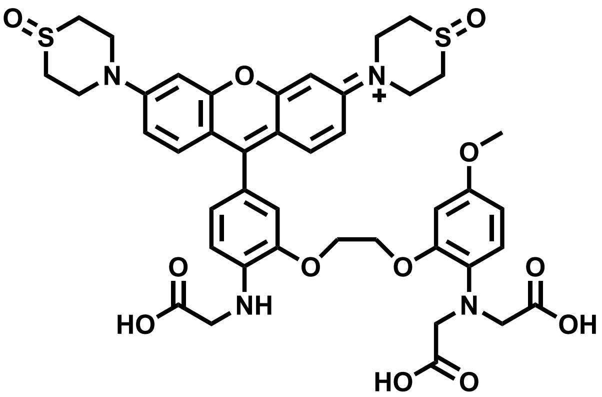

PKZnBR-4 This compound was synthesized according to **General procedure B** and the "PKZnBR dye ester form" mentioned in it referred to **PKZnBR-4 TME** in this part. Yield: 3% over 3 steps (3 mg).

^{1}HNMR (600 MHz, DMSO-d_6) □ 7.71 (d, J = 9.6 Hz, 2H), 7.41 (dd, J = 9.7 Hz, 2.2 Hz, 2H), 7.30 (d, J = 2.2 Hz, 2H), 7.14 (d, J = 1.5 Hz, 1H), 7.06 (dd, J = 8.1 Hz, 1.5 Hz, 2H), 6.78 (d, J = 8.9 Hz, 1H), 6.74 (d, J = 8.4 Hz, 1H), 6.60 (d, J = 2.9 Hz, 1H), 6.45 (dd, J = 8.7 Hz, 2.7 Hz, 1H), 4.39 (s, 4H), 4.26 (d, J = 14.6 Hz, 4H), 4.14 (t, J = 13.2 Hz, 4H), 4.02 (s, 2H), 3.96 (s, 4H), 3.68 (s, 3H), 3.05 ~ 3.01 (m, 4H), 2.88 (d, J = 13.3 Hz, 4H).

^{13}CNMR (151 MHz, DMSO-d_6) □ 172.5, 171.9, 158.6, 157.8, 155.6, 154.9, 151.0, 145.0, 140.8, 132.8, 132.7, 125.4, 120.3, 118.2, 115.0, 113.5, 113.4, 109.1, 105.2, 101.9, 97.8, 67.14, 67.06, 55.3, 53.8, 44.6, 44.1, 40.0.

HRMS (ESI) calculated for $C_{42}H_{45}N_4O_{12}S_2^+$ $[M]^+$ 861.2470, found 861.2475.

NMR Spectra.

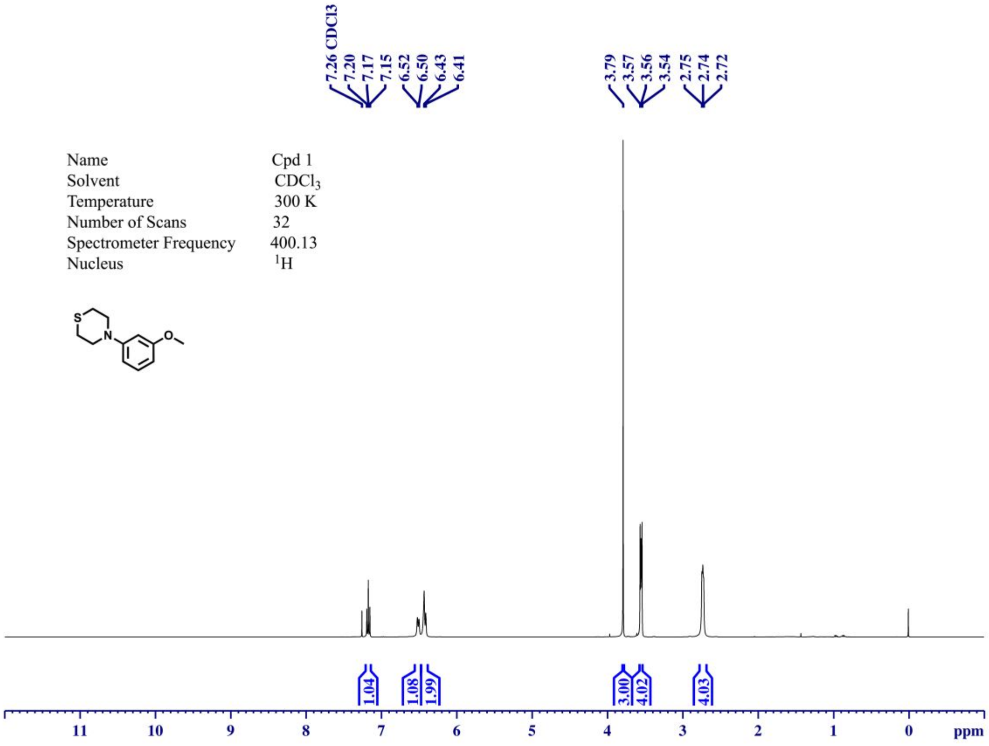
7.26 CDCl3
7.20
7.17
7.15
6.52
6.50
6.43
6.41
3.79
3.57
3.56
3.54
2.75
2.74
2.72
Name Cpd 1
Solvent CDCl3
Temperature 300 K
Number of Scans 32
Spectrometer Frequency 400.13
Nucleus 1H
S
N
O
1.04
1.08
1.99
3.00
4.02
4.03
11 10 9 8 7 6 5 4 3 2 1 0 ppm

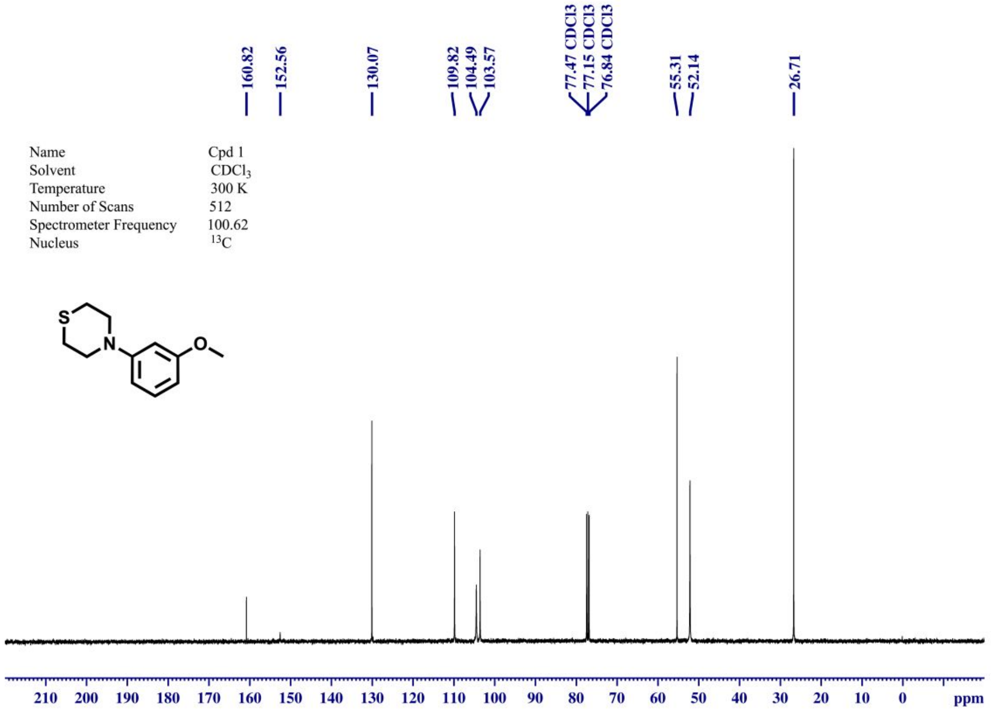
160.82
152.56
130.07
109.82
104.49
103.57
77.47 CDCl3
77.15 CDCl3
76.84 CDCl3
55.31
52.14
26.71
Name Cpd 1
Solvent CDCl3
Temperature 300 K
Number of Scans 512
Spectrometer Frequency 100.62
Nucleus 13C
S
N
O
210 200 190 180 170 160 150 140 130 120 110 100 90 80 70 60 50 40 30 20 10 0 ppm

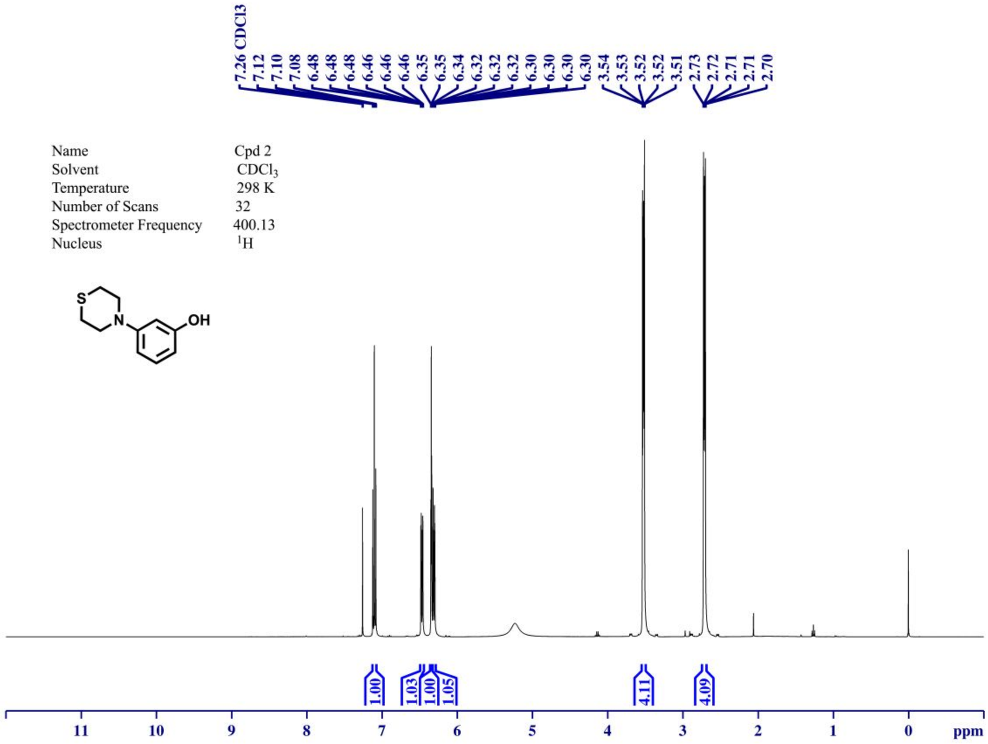
7.26 CDCl3
7.12
7.10
7.08
6.48
6.48
6.48
6.46
6.46
6.35
6.35
6.34
6.32
6.32
6.30
6.30
6.30
6.30
3.54
3.53
3.52
3.52
3.51
2.73
2.72
2.71
2.71
2.70
Name Cpd 2
Solvent CDCl3
Temperature 298 K
Number of Scans 32
Spectrometer Frequency 400.13
Nucleus 1H
S
N
OH
1.00
1.03
1.00
1.05
4.11
4.09
11 10 9 8 7 6 5 4 3 2 1 0 ppm

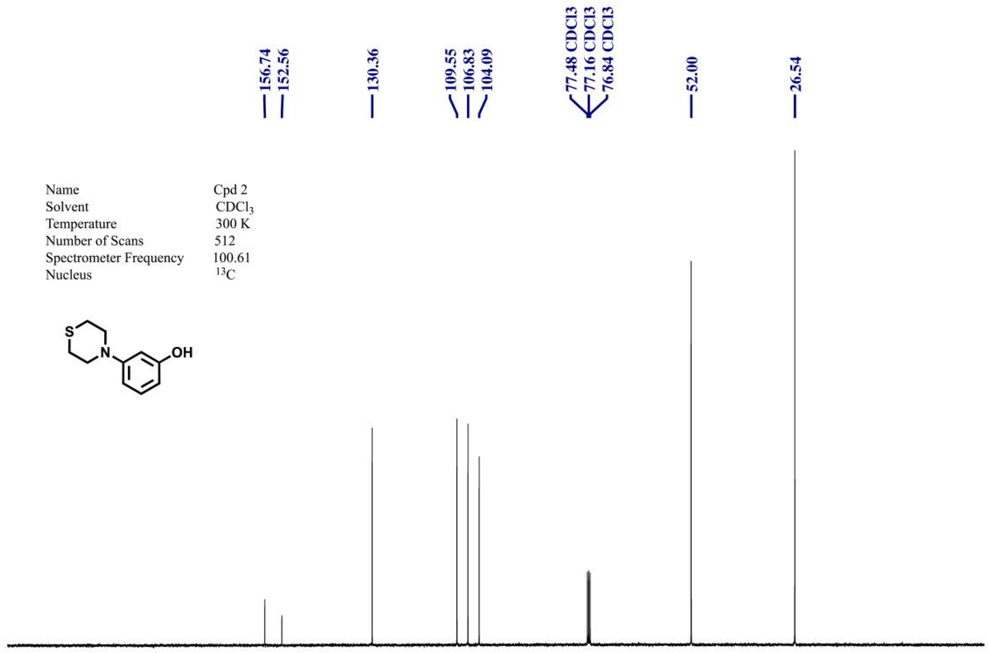
156.74
152.56
130.36
109.55
106.83
104.09
77.48 CDCl3
77.16 CDCl3
76.84 CDCl3
52.00
26.54
Name Cpd 2
Solvent CDCl3
Temperature 300 K
Number of Scans 512
Spectrometer Frequency 100.61
Nucleus 13C
S
N
OH
210 200 190 180 170 160 150 140 130 120 110 100 90 80 70 60 50 40 30 20 10 0 ppm

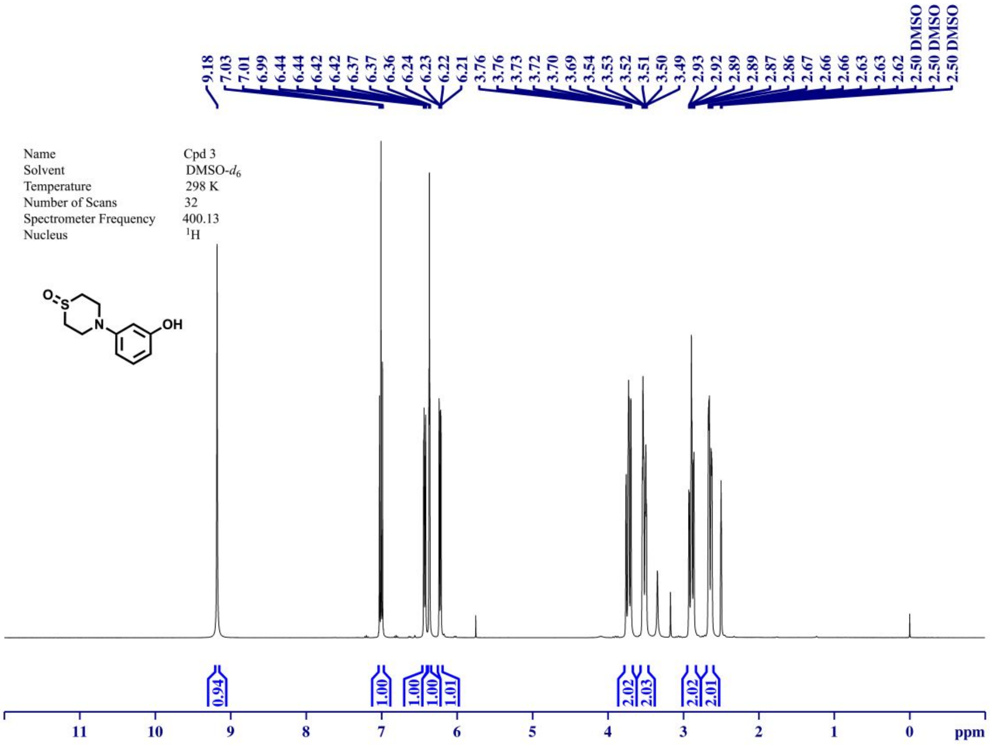
9.18
7.03
7.01
6.99
6.44
6.44
6.42
6.42
6.37
6.37
6.36
6.24
6.23
6.22
6.21
3.76
3.76
3.73
3.72
3.70
3.69
3.54
3.53
3.52
3.51
3.50
3.49
2.93
2.92
2.89
2.89
2.87
2.86
2.67
2.66
2.63
2.63
2.62
2.50 DMSO
2.50 DMSO
2.50 DMSO
Name Cpd 3
Solvent DMSO-d_6
Temperature 298 K
Number of Scans 32
Spectrometer Frequency 400.13
Nucleus ^1H
O
S
N
OH
0.94
1.00
1.00
1.00
1.01
2.02
2.03
2.02
2.01
11 10 9 8 7 6 5 4 3 2 1 0 ppm

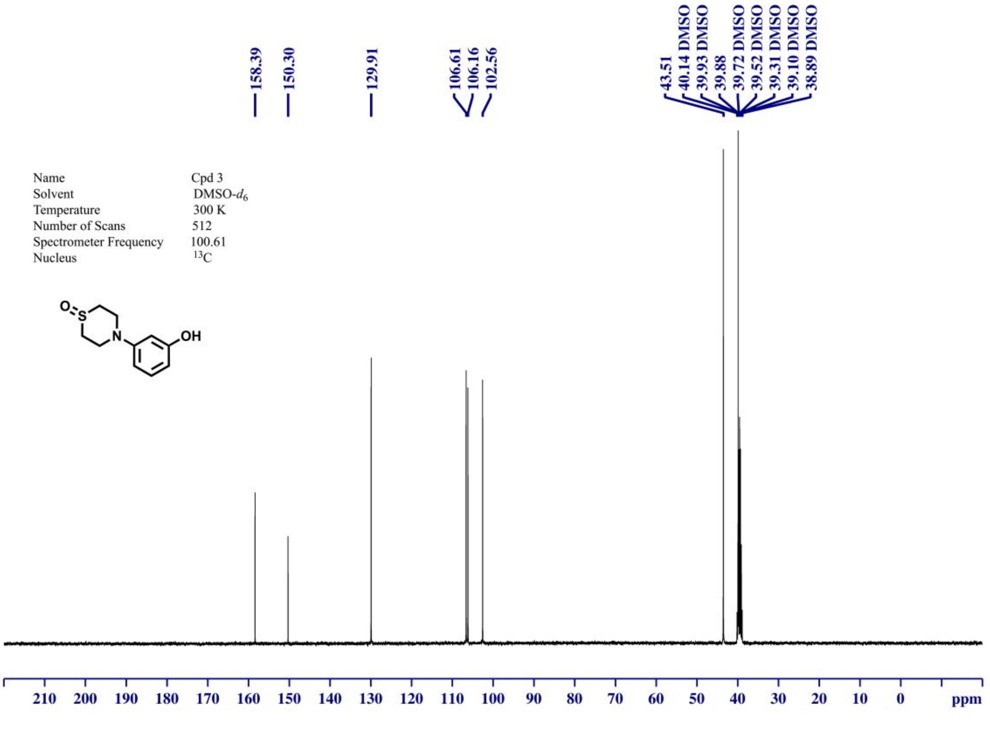
158.39
150.30
129.91
106.61
106.16
102.56
43.51
40.14 DMSO
39.93 DMSO
39.88
39.72 DMSO
39.52 DMSO
39.31 DMSO
39.10 DMSO
38.89 DMSO
Name Cpd 3
Solvent DMSO-d_6
Temperature 300 K
Number of Scans 512
Spectrometer Frequency 100.61
Nucleus ^{13}C
O
S
N
OH
210 200 190 180 170 160 150 140 130 120 110 100 90 80 70 60 50 40 30 20 10 0 ppm

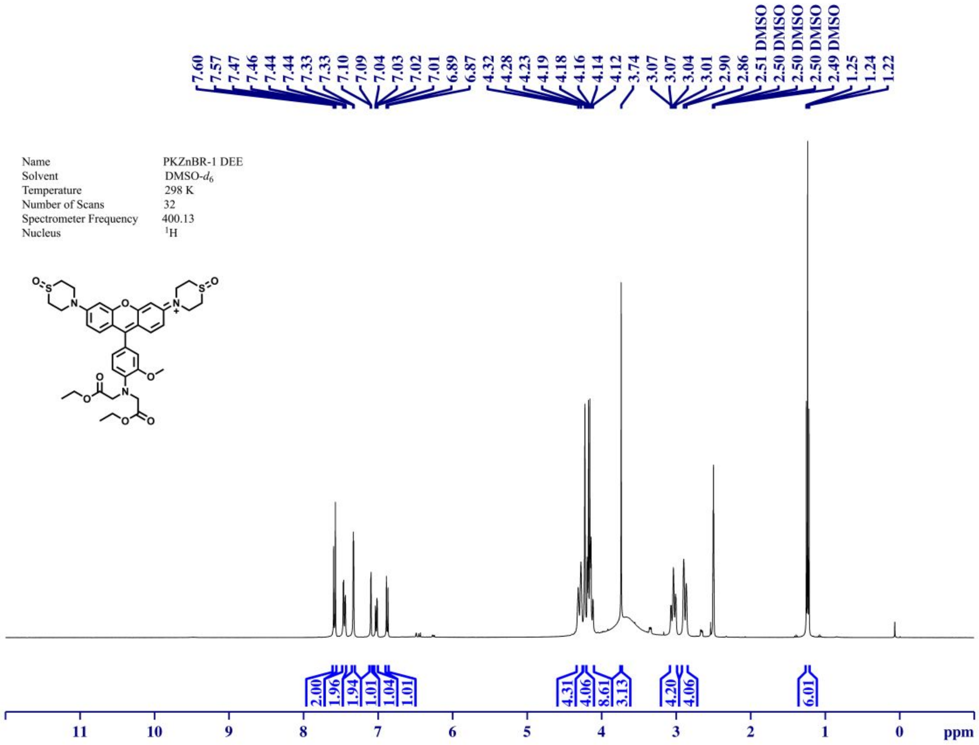
7.60 7.57 7.47 7.46 7.44 7.44 7.33 7.33 7.10 7.09 7.04 7.03 7.02 7.01 6.89 6.87 4.32 4.28 4.23 4.19 4.18 4.16 4.14 4.12 3.74 3.07 3.07 3.04 3.01 2.90 2.86 2.51 DMSO 2.50 DMSO 2.50 DMSO 2.50 DMSO 2.49 DMSO 1.25 1.24 1.22
Name PKZnBR-1 DEE
Solvent DMSO-d_6
Temperature 298 K
Number of Scans 32
Spectrometer Frequency 400.13
Nucleus ^{1}H
2.00 1.96 1.94 1.01 1.04 1.01 4.31 4.06 8.61 3.13 4.20 4.06 6.01
11 10 9 8 7 6 5 4 3 2 1 0 ppm

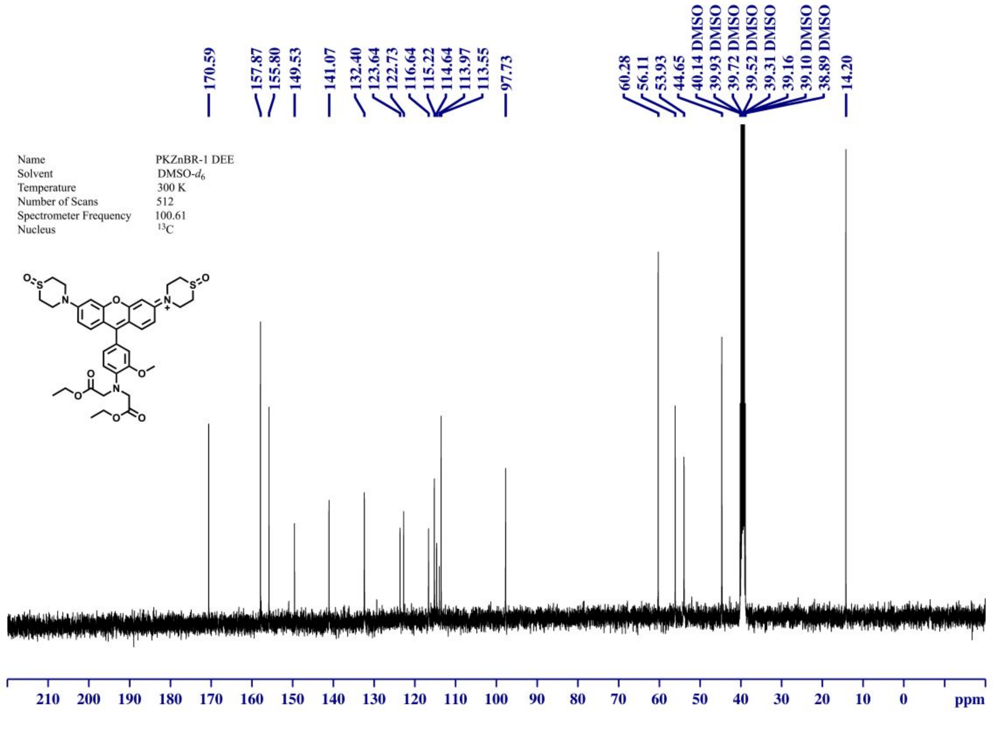
170.59 157.87 155.80 149.53 141.07 132.40 123.64 122.73 116.64 115.22 114.64 113.97 113.55 97.73 60.28 56.11 53.93 44.65 40.14 DMSO 39.93 DMSO 39.72 DMSO 39.52 DMSO 39.31 DMSO 39.16 39.10 DMSO 38.89 DMSO 14.20
Name PKZnBR-1 DEE
Solvent DMSO-d_6
Temperature 300 K
Number of Scans 512
Spectrometer Frequency 100.61
Nucleus ^{13}C
210 200 190 180 170 160 150 140 130 120 110 100 90 80 70 60 50 40 30 20 10 0 ppm

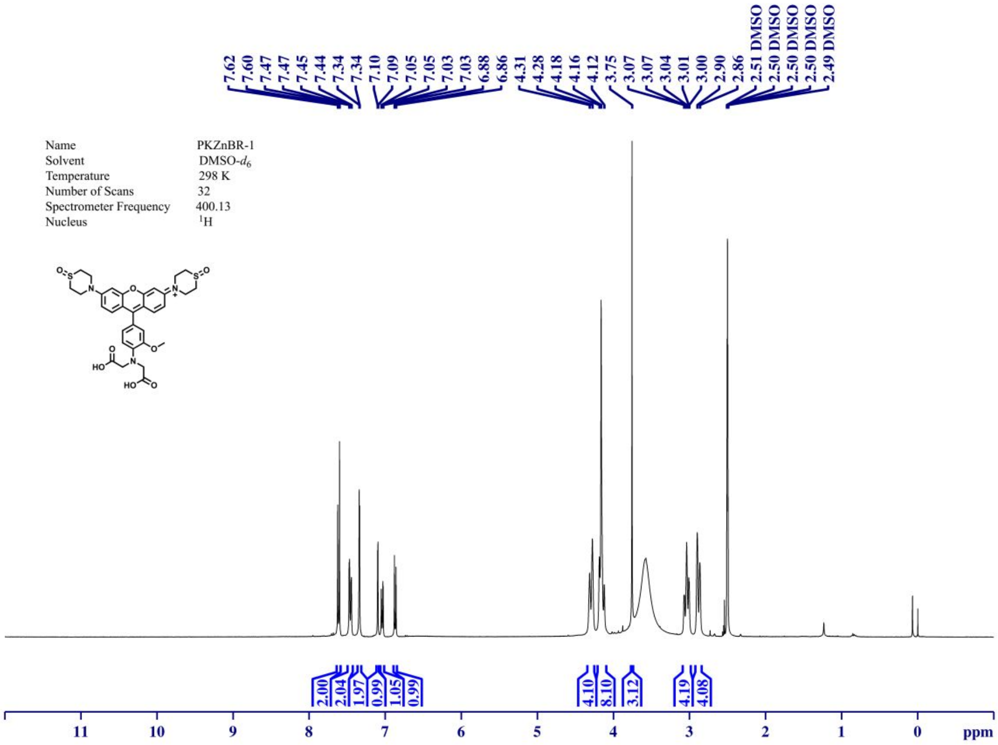
7.62 7.60 7.47 7.47 7.45 7.44 7.34 7.34 7.10 7.09 7.05 7.05 7.03 7.03 6.88 6.86 4.31 4.28 4.18 4.16 4.12 3.75 3.07 3.07 3.04 3.01 3.00 2.90 2.86 2.51 DMSO 2.50 DMSO 2.50 DMSO 2.50 DMSO 2.49 DMSO
Name PKZnBR-1
Solvent DMSO-d_6
Temperature 298 K
Number of Scans 32
Spectrometer Frequency 400.13
Nucleus ^{1}H
2.00 2.04 1.97 0.99 1.05 0.99 4.10 8.10 3.12 4.19 4.08
11 10 9 8 7 6 5 4 3 2 1 0 ppm

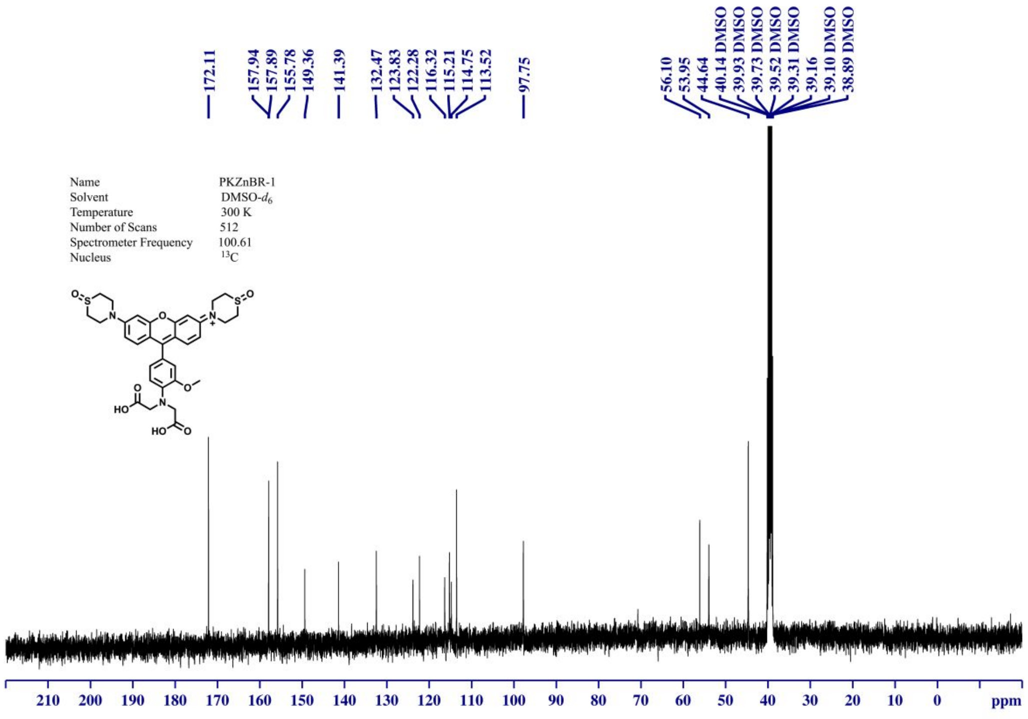
172.11 157.94 157.89 155.78 149.36 141.39 132.47 123.83 122.28 116.32 115.21 114.75 113.52 97.75 56.10 53.95 44.64 40.14 DMSO 39.93 DMSO 39.73 DMSO 39.52 DMSO 39.31 DMSO 39.16 39.10 DMSO 38.89 DMSO
Name PKZnBR-1
Solvent DMSO-d_6
Temperature 300 K
Number of Scans 512
Spectrometer Frequency 100.61
Nucleus ^{13}C
210 200 190 180 170 160 150 140 130 120 110 100 90 80 70 60 50 40 30 20 10 0 ppm

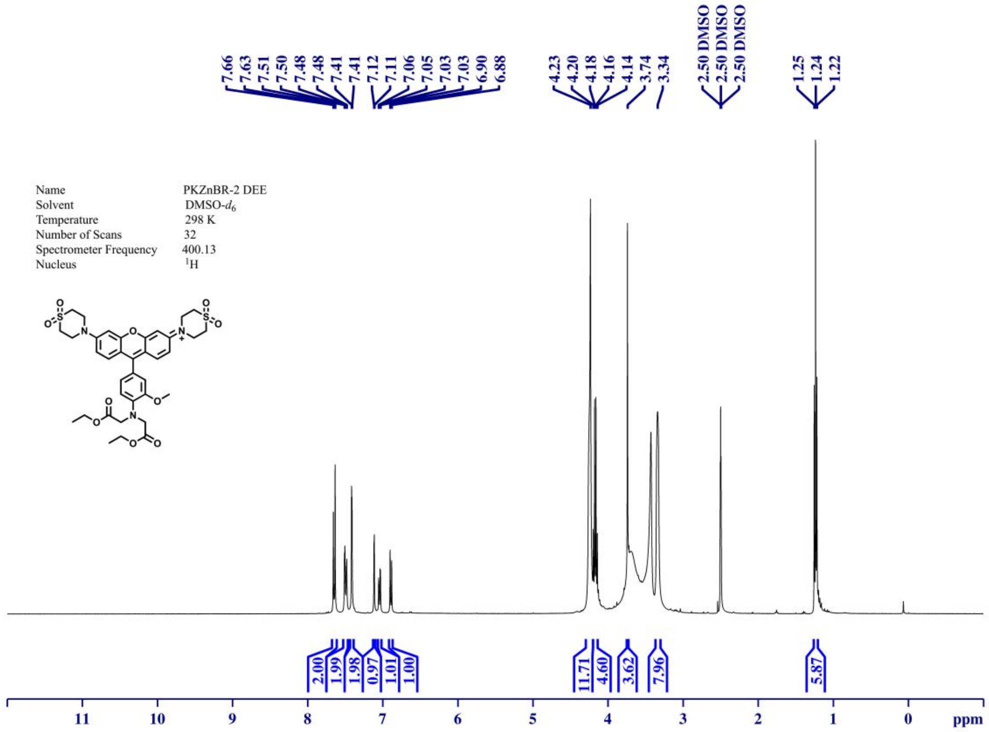
Name PKZnBR-2 DEE
Solvent DMSO-d_6
Temperature 298 K
Number of Scans 32
Spectrometer Frequency 400.13
Nucleus ^{1}H
7.66 7.63 7.51 7.50 7.48 7.48 7.41 7.41 7.12 7.11 7.06 7.05 7.03 7.03 6.90 6.88
4.23 4.20 4.18 4.16 4.14 3.74 3.34
2.50 DMSO 2.50 DMSO 2.50 DMSO
1.25 1.24 1.22
2.00 1.99 1.98 0.97 1.01 1.00 11.71 4.60 3.62 7.96 5.87
11 10 9 8 7 6 5 4 3 2 1 0 ppm

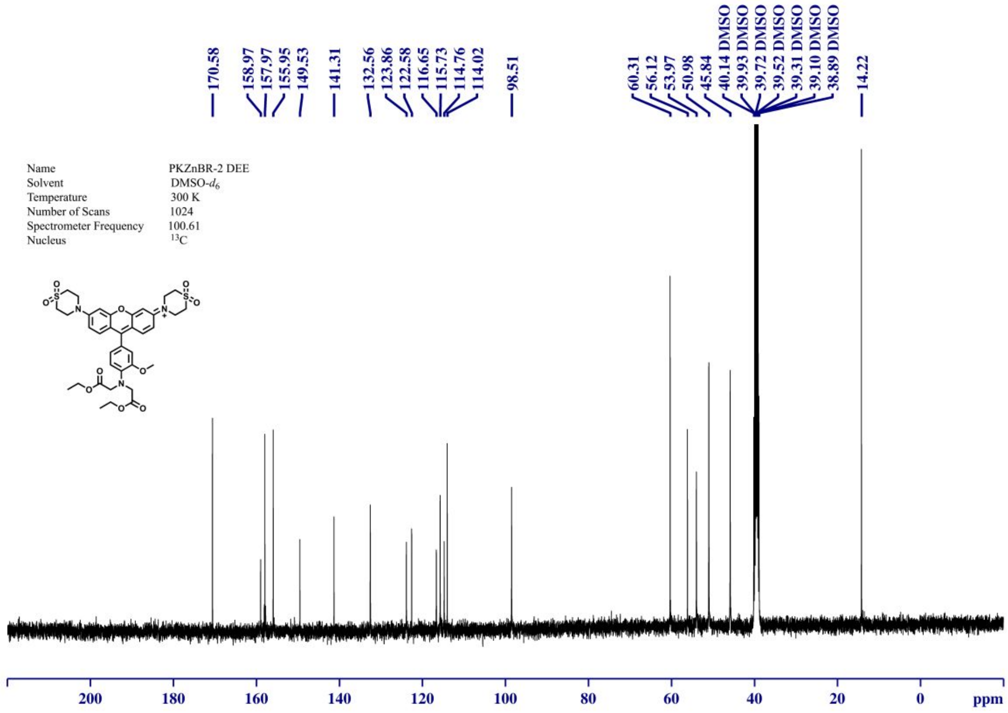
Name PKZnBR-2 DEE
Solvent DMSO-d_6
Temperature 300 K
Number of Scans 1024
Spectrometer Frequency 100.61
Nucleus ^{13}C
170.58
158.97 157.97 155.95 149.53
141.31
132.56 123.86 122.58 116.65 115.73 114.76 114.02
98.51
60.31 56.12 53.97 50.98 45.84
40.14 DMSO 39.93 DMSO 39.72 DMSO 39.52 DMSO 39.31 DMSO 39.10 DMSO 38.89 DMSO
14.22
200 180 160 140 120 100 80 60 40 20 0 ppm

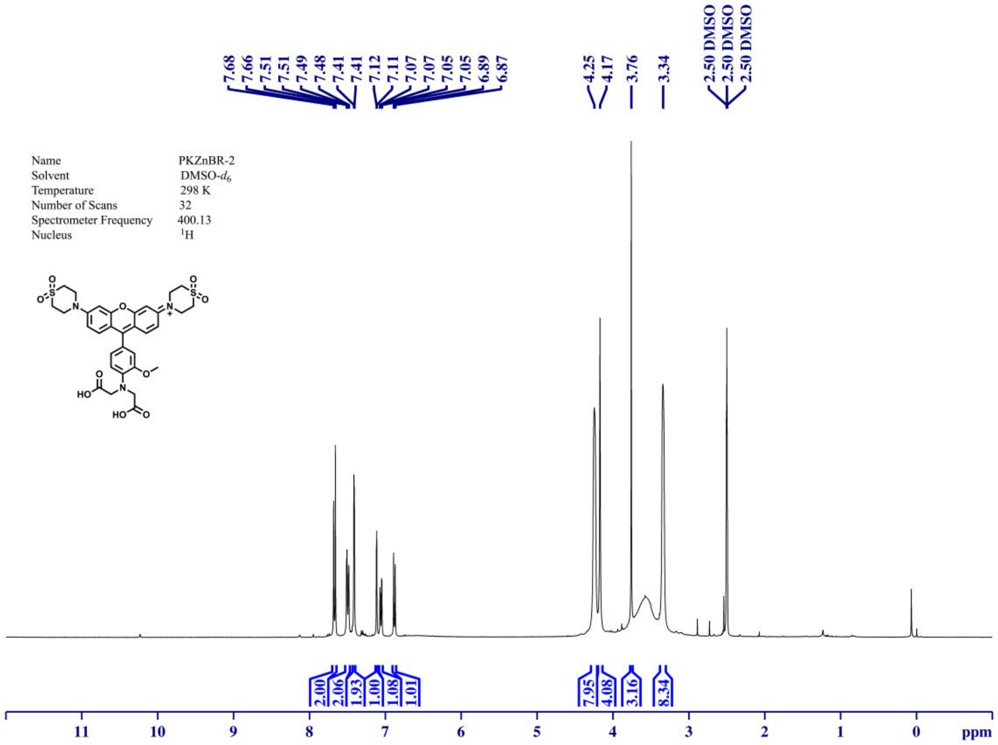
7.68
7.66
7.51
7.51
7.49
7.48
7.41
7.41
7.12
7.11
7.07
7.07
7.05
7.05
6.89
6.87
4.25
4.17
3.76
3.34
2.50 DMSO
2.50 DMSO
2.50 DMSO
Name PKZnBR-2
Solvent DMSO-d_6
Temperature 298 K
Number of Scans 32
Spectrometer Frequency 400.13
Nucleus ^{1}H
2.00
2.06
1.93
1.00
1.08
1.01
7.95
4.08
3.16
8.34
11
10
9
8
7
6
5
4
3
2
1
0
ppm

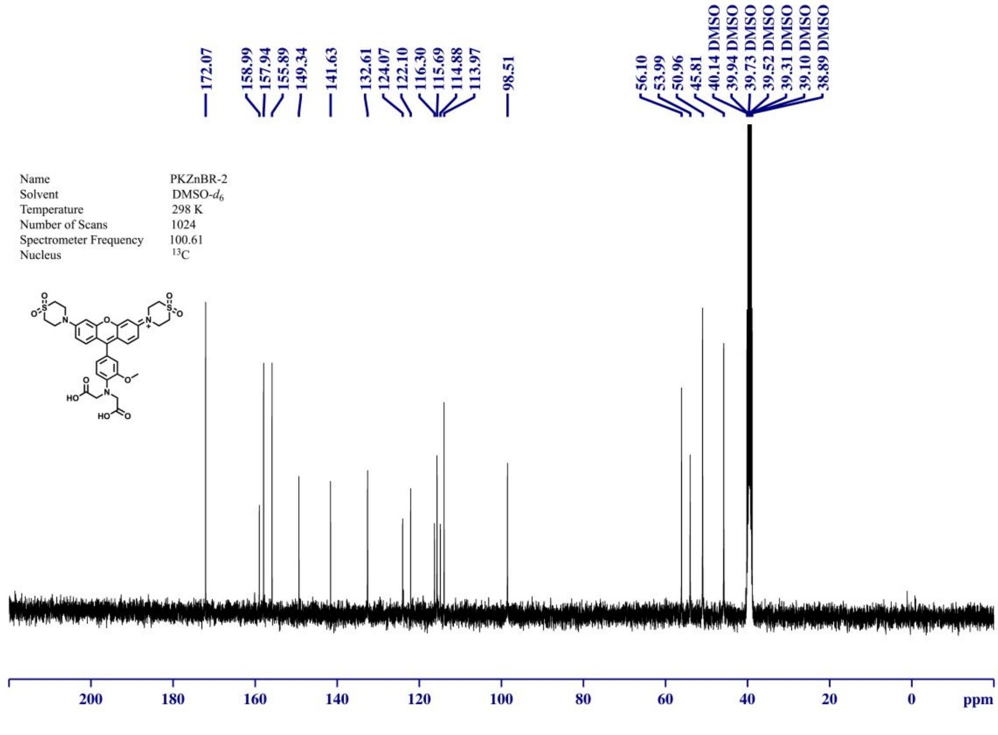
172.07
158.99
157.94
155.89
149.34
141.63
132.61
124.07
122.10
116.30
115.69
114.88
113.97
98.51
56.10
53.99
50.96
45.81
40.14 DMSO
39.94 DMSO
39.73 DMSO
39.52 DMSO
39.31 DMSO
39.10 DMSO
38.89 DMSO
Name PKZnBR-2
Solvent DMSO-d_6
Temperature 298 K
Number of Scans 1024
Spectrometer Frequency 100.61
Nucleus ^{13}C
200
180
160
140
120
100
80
60
40
20
0
ppm

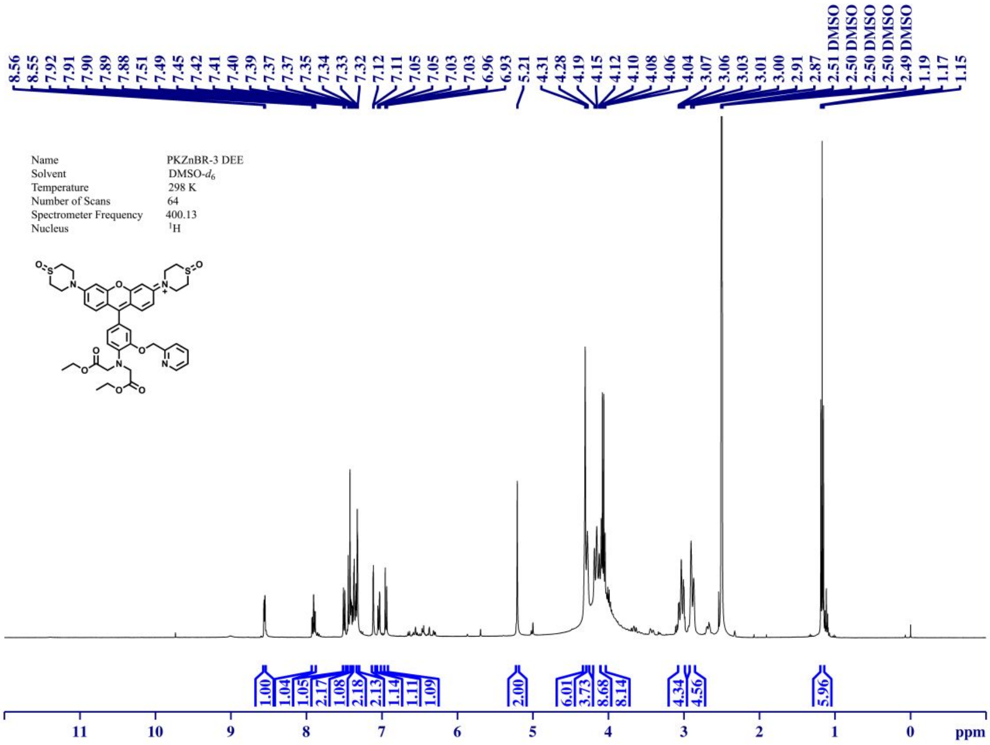
8.56 8.55 7.92 7.91 7.90 7.89 7.88 7.51 7.49 7.45 7.42 7.41 7.40 7.39 7.37 7.37 7.35 7.34 7.33 7.32 7.12 7.11 7.05 7.05 7.03 7.03 6.96 6.93 5.21 4.31 4.28 4.19 4.15 4.12 4.10 4.08 4.06 4.04 3.07 3.06 3.03 3.01 3.00 2.91 2.87 2.51 DMSO 2.50 DMSO 2.50 DMSO 2.50 DMSO 2.49 DMSO 1.19 1.17 1.15
Name PKZnBR-3 DEE
Solvent DMSO-d_6
Temperature 298 K
Number of Scans 64
Spectrometer Frequency 400.13
Nucleus ^{1}H
1.00 1.04 1.05 2.17 1.08 2.18 2.13 1.14 1.11 1.09 2.00 6.01 3.73 8.68 8.14 4.34 4.56 5.96
11 10 9 8 7 6 5 4 3 2 1 0 ppm

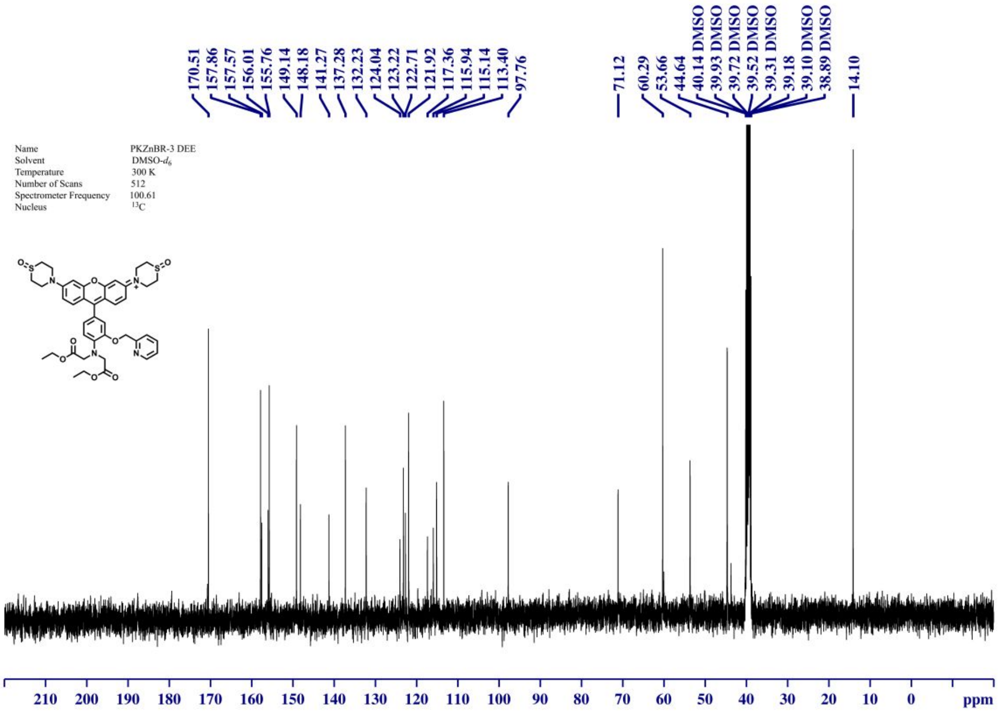
170.51 157.86 157.57 156.01 155.76 149.14 148.18 141.27 137.28 132.23 124.04 123.22 122.71 121.92 117.36 115.94 115.14 113.40 97.76 71.12 60.29 53.66 44.64 40.14 DMSO 39.93 DMSO 39.72 DMSO 39.52 DMSO 39.31 DMSO 39.18 39.10 DMSO 38.89 DMSO 14.10
Name PKZnBR-3 DEE
Solvent DMSO-d_6
Temperature 300 K
Number of Scans 512
Spectrometer Frequency 100.61
Nucleus ^{13}C
210 200 190 180 170 160 150 140 130 120 110 100 90 80 70 60 50 40 30 20 10 0 ppm

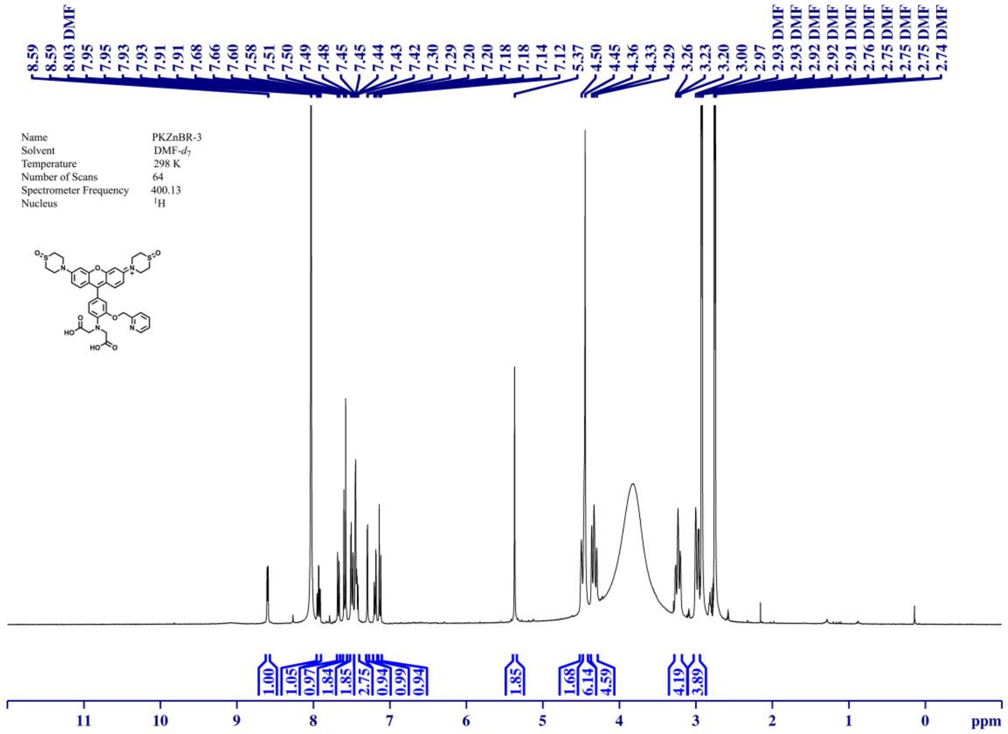
8.59 8.59 8.03 DMF 7.95 7.95 7.93 7.93 7.91 7.91 7.68 7.66 7.60 7.58 7.51 7.50 7.49 7.48 7.45 7.45 7.44 7.43 7.42 7.30 7.29 7.20 7.20 7.18 7.14 7.12 5.37 4.50 4.45 4.36 4.33 4.29 3.26 3.23 3.20 3.00 2.97 2.93 DMF 2.92 DMF 2.92 DMF 2.91 DMF 2.76 DMF 2.75 DMF 2.75 DMF 2.74 DMF
Name PKZnBR-3
Solvent DMF-d_7
Temperature 298 K
Number of Scans 64
Spectrometer Frequency 400.13
Nucleus ^{1}H
1.00 1.05 0.97 1.84 1.85 2.75 0.94 0.99 0.94 1.85 1.68 6.14 4.59 4.19 3.89
11 10 9 8 7 6 5 4 3 2 1 0 ppm

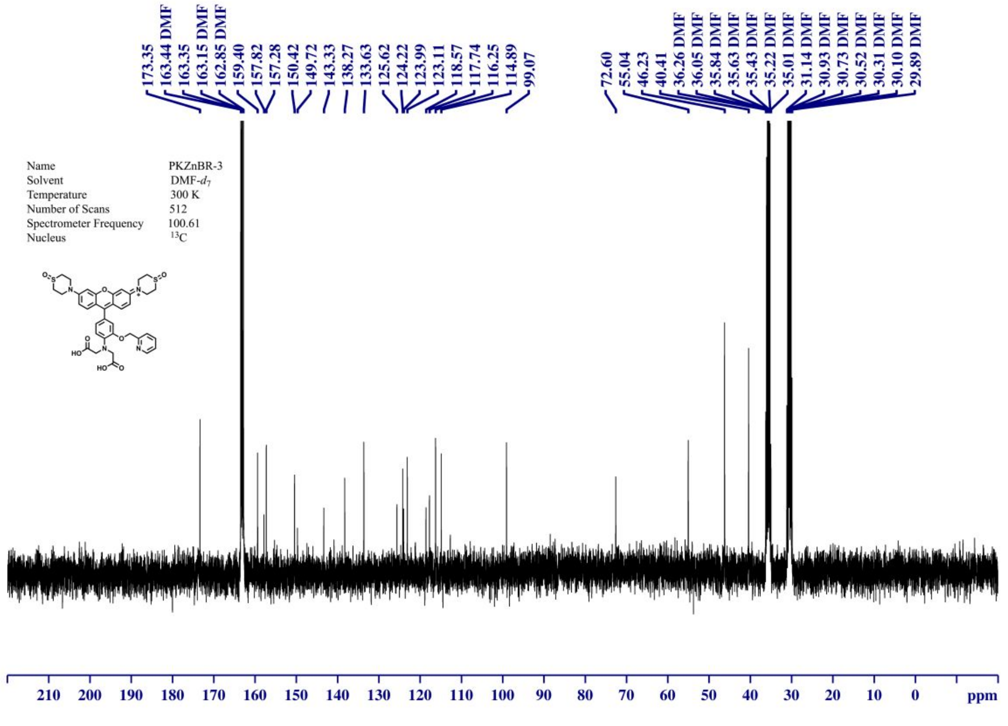
173.35 163.44 DMF 163.35 163.15 DMF 162.85 DMF 159.40 157.82 157.28 150.42 149.72 143.33 138.27 133.63 125.62 124.22 123.99 123.11 118.57 117.74 116.25 114.89 99.07 72.60 55.04 46.23 40.41 36.26 DMF 36.05 DMF 35.84 DMF 35.63 DMF 35.43 DMF 35.22 DMF 35.01 DMF 31.14 DMF 30.93 DMF 30.73 DMF 30.52 DMF 30.31 DMF 30.10 DMF 29.89 DMF
Name PKZnBR-3
Solvent DMF-d_7
Temperature 300 K
Number of Scans 512
Spectrometer Frequency 100.61
Nucleus ^{13}C
210 200 190 180 170 160 150 140 130 120 110 100 90 80 70 60 50 40 30 20 10 0 ppm

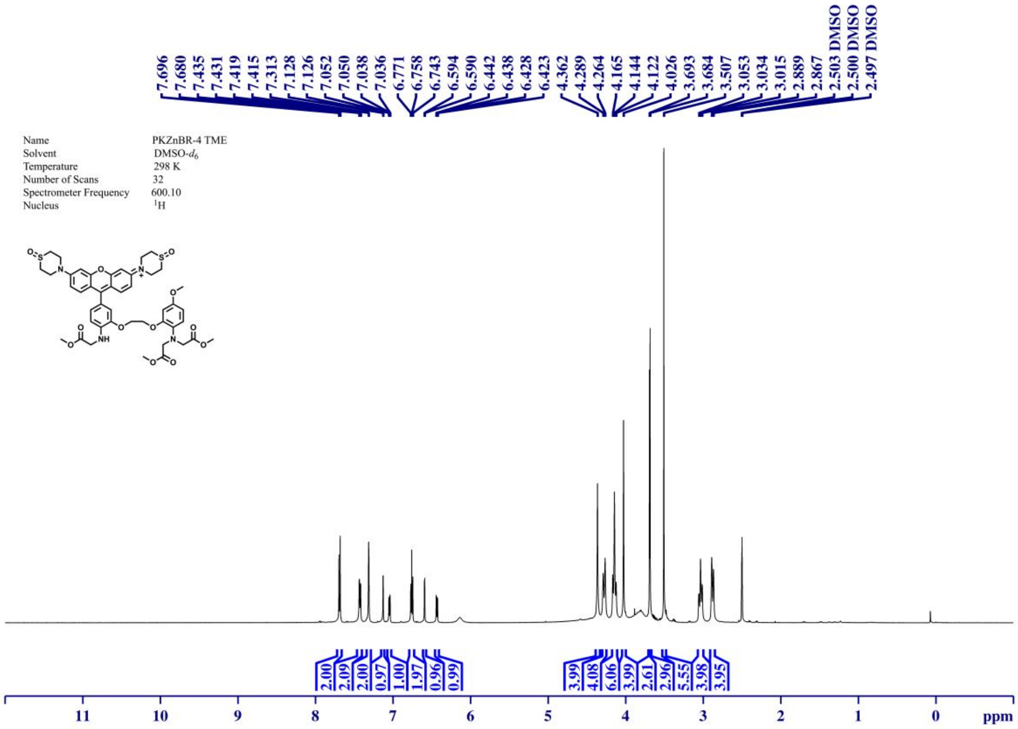
7.696
7.680
7.435
7.431
7.419
7.415
7.313
7.128
7.126
7.052
7.050
7.038
7.036
6.771
6.758
6.743
6.594
6.590
6.442
6.438
6.428
6.423
4.362
4.289
4.264
4.165
4.144
4.122
4.026
3.693
3.684
3.507
3.053
3.034
3.015
2.889
2.867
2.503 DMSO
2.500 DMSO
2.497 DMSO
Name PKZnBR-4 TME
Solvent DMSO-d_6
Temperature 298 K
Number of Scans 32
Spectrometer Frequency 600.10
Nucleus ^{1}H
NH
2.00
2.09
2.00
0.97
1.00
1.97
0.96
0.99
3.99
4.08
6.06
3.99
2.61
2.96
5.55
3.98
3.95
11
10
9
8
7
6
5
4
3
2
1
0
ppm

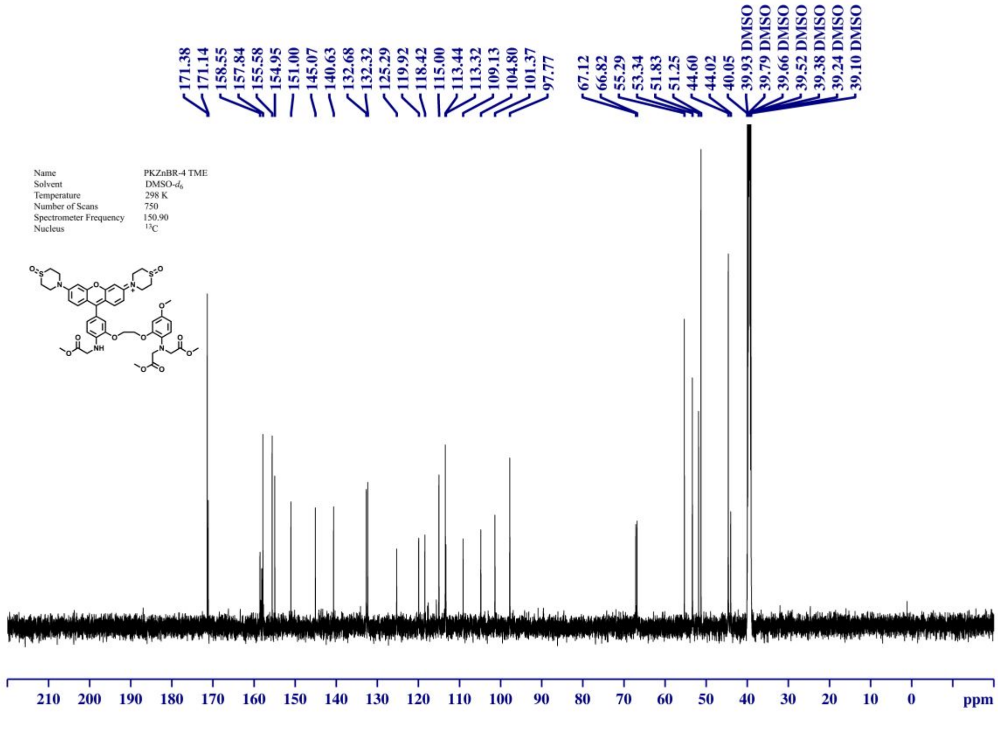
171.38
171.14
158.55
157.84
155.58
154.95
151.00
145.07
140.63
132.68
132.32
125.29
119.92
118.42
115.00
113.44
113.32
109.13
104.80
101.37
97.77
67.12
66.82
55.29
53.34
51.83
51.25
44.60
44.02
40.05
39.93 DMSO
39.79 DMSO
39.66 DMSO
39.52 DMSO
39.38 DMSO
39.24 DMSO
39.10 DMSO
Name PKZnBR-4 TME
Solvent DMSO-d_6
Temperature 298 K
Number of Scans 750
Spectrometer Frequency 150.90
Nucleus ^{13}C
NH
210
200
190
180
170
160
150
140
130
120
110
100
90
80
70
60
50
40
30
20
10
0
ppm

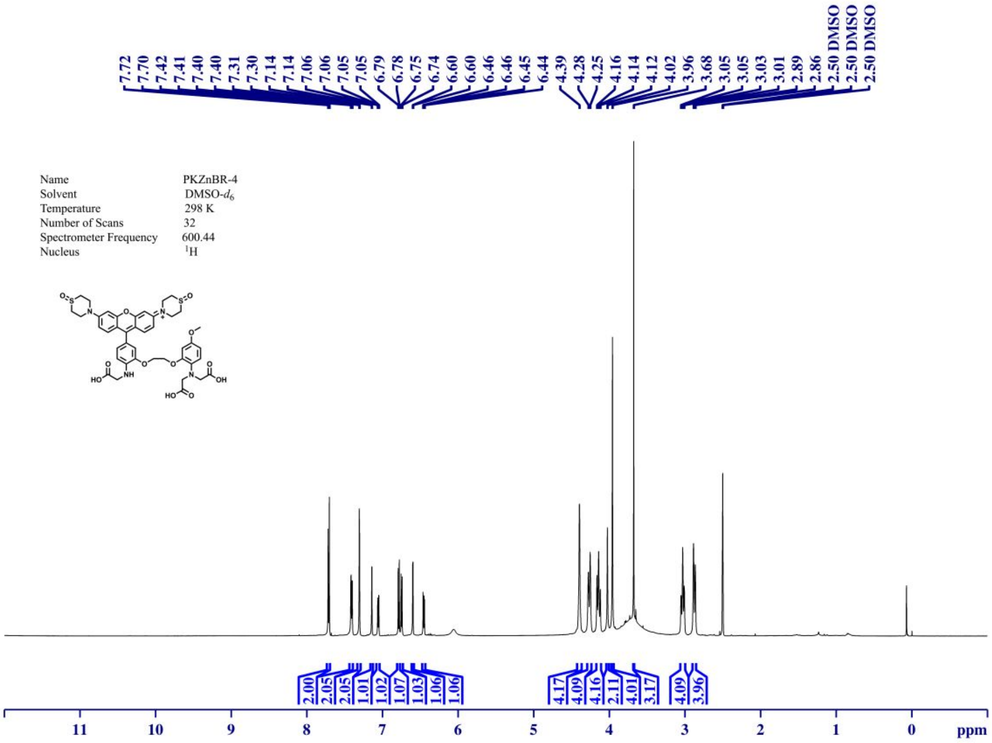
7.72
7.70
7.42
7.41
7.40
7.40
7.31
7.30
7.14
7.14
7.06
7.06
7.05
7.05
6.79
6.78
6.75
6.74
6.60
6.46
6.46
6.45
6.44
4.39
4.28
4.25
4.16
4.14
4.12
4.02
3.96
3.68
3.05
3.05
3.03
3.01
2.89
2.86
2.50 DMSO
2.50 DMSO
2.50 DMSO
Name PKZnBR-4
Solvent DMSO-d_6
Temperature 298 K
Number of Scans 32
Spectrometer Frequency 600.44
Nucleus ^{1}H
2.00
2.05
2.05
1.01
1.02
1.07
1.03
1.06
1.06
4.17
4.09
4.16
2.11
4.01
3.17
4.09
3.96
11
10
9
8
7
6
5
4
3
2
1
0
ppm

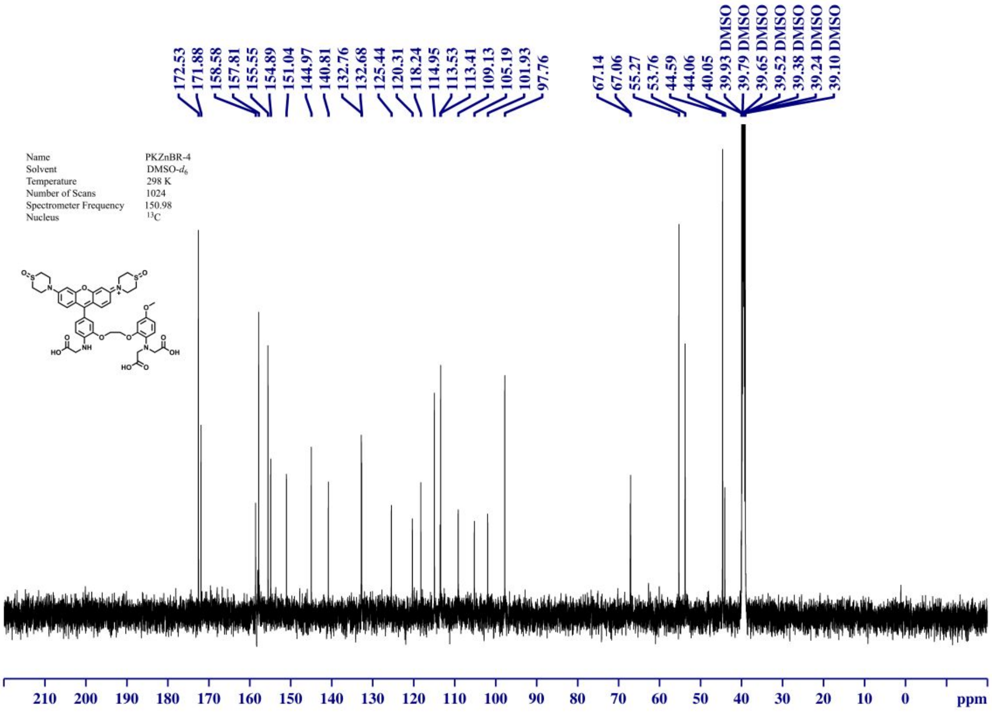
172.53
171.88
158.58
157.81
155.55
154.89
151.04
144.97
140.81
132.76
132.68
125.44
120.31
118.24
114.95
113.53
113.41
109.13
105.19
101.93
97.76
67.14
67.06
55.27
53.76
44.59
44.06
40.05
39.93 DMSO
39.79 DMSO
39.65 DMSO
39.52 DMSO
39.38 DMSO
39.24 DMSO
39.10 DMSO
Name PKZnBR-4
Solvent DMSO-d_6
Temperature 298 K
Number of Scans 1024
Spectrometer Frequency 150.98
Nucleus ^{13}C
210
200
190
180
170
160
150
140
130
120
110
100
90
80
70
60
50
40
30
20
10
0
ppm

References

(1) Zhang, J.; Peng, X.; Wu, Y.; Ren, H.; Sun, J.; Tong, S.; Liu, T.; Zhao, Y.; Wang, S.; Tang, C.; Chen, L.; Chen, Z., Red- and Far-Red-Emitting Zinc Probes with Minimal Phototoxicity for Multiplexed Recording of Orchestrated Insulin Secretion. *Angewandte Chemie International Edition* **2021,** *60*, 25846-25855.

(2) Iyoshi, S.; Taki, M.; Yamamoto, Y., Development of a Cholesterol-Conjugated Fluorescent Sensor for Site-Specific Detection of Zinc Ion at the Plasma Membrane. *Organic Letters* **2011,** *13*, 4558-4561.

(3) Sensi, S. L.; Ton-That, D.; Weiss, J. H.; Rothe, A.; Gee, K. R., A new mitochondrial fluorescent zinc sensor. *Cell Calcium* **2003,** *34*, 281-284.

(4) Gee, K. R.; Zhou, Z.-L.; Qian, W.-J.; Kennedy, R., Detection and Imaging of Zinc Secretion from Pancreatic β-Cells Using a New Fluorescent Zinc Indicator. *J Am Chem Soc* **2002,** *124*, 776-778.
